# Nanoprogrammed Cross-Kingdom Communication Between Living Microorganisms

**DOI:** 10.1021/acs.nanolett.1c02435

**Published:** 2022-02-16

**Authors:** Beatriz de Luis, Ángela Morellá-Aucejo, Antoni Llopis-Lorente, Javier Martínez-Latorre, Félix Sancenón, Carmelo López, José Ramón Murguía, Ramón Martínez-Máñez

**Affiliations:** † Instituto Interuniversitario de Investigación de Reconocimiento Molecular y Desarrollo Tecnológico (IDM), Universitat Politècnica de València, Universitat de València, Camino de Vera s/n, 46022 Valencia, Spain; ‡ CIBER de Bioingeniería, Biomateriales y Nanomedicina (CIBER-BBN), 28029 Madrid, Spain; § Unidad Mixta UPV-CIPF de Investigación en Mecanismos de Enfermedades y Nanomedicina, Centro de Investigación Príncipe Felipe, Universitat Politècnica de València, 46012 Valencia, Spain; ∥ Unidad Mixta de Investigación en Nanomedicina y Sensores, Instituto de Investigación Sanitaria La Fe, Universitat Politècnica de València, 46026 Valencia, Spain; ⊥ Instituto Universitario de Conservación y Mejora de la Agrodiversidad Valenciana, Universitat Politècnica de València (COMAV-UPV), 46022 Valencia, Spain

**Keywords:** chemical communication, nanotranslator, microorganisms, nanonetworks, cross-kingdom, cell communication

## Abstract

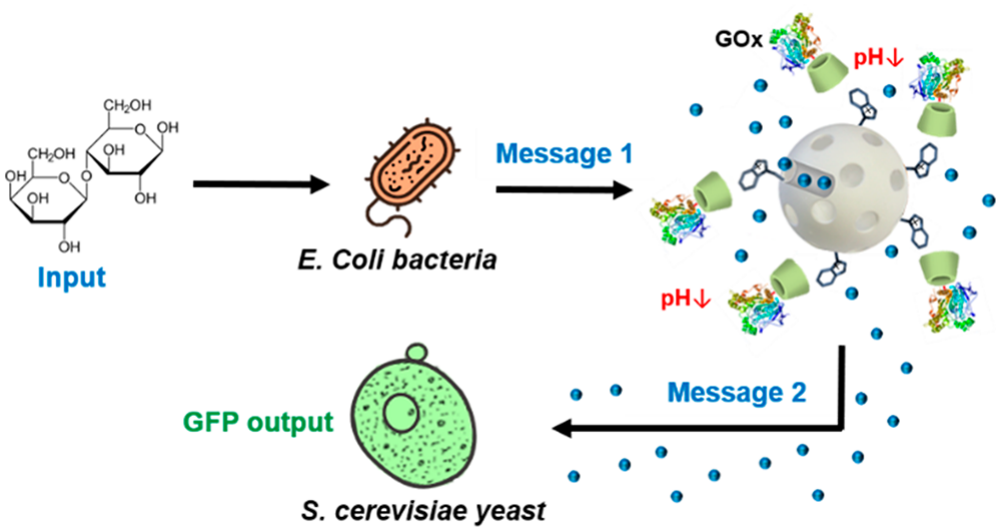

The engineering of chemical communication at the micro/nanoscale is a key emergent topic in micro/nanotechnology, synthetic biology, and related areas. However, the field is still in its infancy; previous advances, although scarce, have mainly focused on communication between abiotic micro/nanosystems or between microvesicles and living cells. Here, we have implemented a nanoprogrammed cross-kingdom communication involving two different microorganisms and tailor-made nanodevices acting as “nanotranslators”. Information flows from the sender cells (bacteria) to the nanodevice and from the nanodevice to receiver cells (yeasts) in a hierarchical way, allowing communication between two microorganisms that otherwise would not interact.

Living systems react to molecular signals in their environment via evolved biochemical sensory pathways that determine their adaptability, function, and survival.^1−3^ Moreover, chemical communication routes allow sharing information between peers and the orchestration of collective behaviors.^4−6^ For instance, bacteria communicate via quorum sensing, that is, individuals release signaling molecules (the so-called autoinducers or quorum molecules) and upon reaching a threshold cell-autoinducer concentration, collective functions (e.g., biofilm formation, virulence, genetic regulation) are activated.^7^ Within a kingdom, organisms use similar pathways to communicate with a member of the same species (i.e., pheromones in the animal kingdom, quorum molecules in the bacteria kingdom, mating factors in fungi, and so forth). In contrast, organisms of different kingdom do not usually communicate; communication is restricted unless a particular cross-kingdom communication pathway has emerged providing a certain advantage during species evolution.^8−10^

The design of chemical communication networks at the micro/nanoscale is an emergent interdisciplinary topic with potential applications in diverse areas such as sensing, biomedicine, biotechnology, and information and communication technologies.^11−13^ In this scenario, despite advances in micro/nanotechnology and synthetic biology^14−16^ most of the micro/nanoparticles reported so far have been studied as single units, whereas the engineering of abiotic micro/nanosystems able to communicate is underexplored and represents a paradigm shift. In communication theory terms, communication involves the transmission of information from a sender to a receiver, that is, the sender channels a message through a suitable medium to be decoded by the receiver.^11,12^ Communication is considered effective if it exerts the desired action on the receiver. This sender–receiver communication between two entities has served as the basis for developing communication systems at the micro/nanoscale. The few studies in this direction can be divided in two main categories: (i) communication between abiotic systems and (ii) communication between living and abiotic systems. Several strategies have been reported to communicate micro/nanoparticles, such as the utility of DNA-strand displacement reactions,^17−20^ enzymatic cascades,^21−24^ and stimuli-responsive delivery systems.^25−28^ Efforts to communicate abiotic with living systems have mainly relied on the incorporation of transcription-translation extracts in microscale compartments (i.e., lipid microvesicles) able to translate molecular information from the environment and/or encapsulated components into a suitable messenger to induce a response in cells.^29−34^ Despite these reported examples, the demonstration of more complex pathways is a requirement to spur advances in the area with the future aim to integrate collectives of nano/microparticles and living systems with advanced functions.

In this context, we present, as a proof-of-concept, to the best of our knowledge the first realization of a programmed cross-kingdom communication involving two species of living cells enabled by tailor-made nanoparticles. In the first place, the engineered scheme comprises communication from the first type of cells to the nanoparticles in response to an external stimulus. Subsequently, the nanoparticles decode the received chemical message and emit a new message detected by the second type of cells which trigger a second response. The overall network can be described as living-to-abiotic-to-living cascade-like communication in which an abiotic nanodevice acts as “nanotranslator” allowing communication between two cells from different kingdoms that otherwise would not interact. In particular, we employed *Escherichia coli* (prokaryotic cells, bacteria kingdom) and *Saccharomyces cerevisiae* (eukaryotic cells, fungi kingdom) as model microorganisms. The “nanotranslator” consists of mesoporous silica nanoparticles loaded with a molecular messenger (phleomycin) and capped with a glucose oxidase (GOx)-based responsive gatekeeper. As illustrated in Scheme 1C, communication is triggered in the presence of lactose (input) which is sensed and hydrolyzed by *E. coli* cells (β-galactosidase-expressing, vide infra) into glucose and galactose. Glucose (first chemical messenger) is then detected by glucose oxidase (GOx) on the abiotic nanodevice, inducing the uncapping of the pH-sensitive gatekeeper and resulting in the release of phleomycin (second chemical messenger). Finally, in response to phleomycin *S. cerevisiae* yeast cells activate a genetic cascade that leads to green fluorescent protein (GFP)^35^ expression and the subsequent production a fluorescence signal as the output of the communication network.

**Scheme 1:**
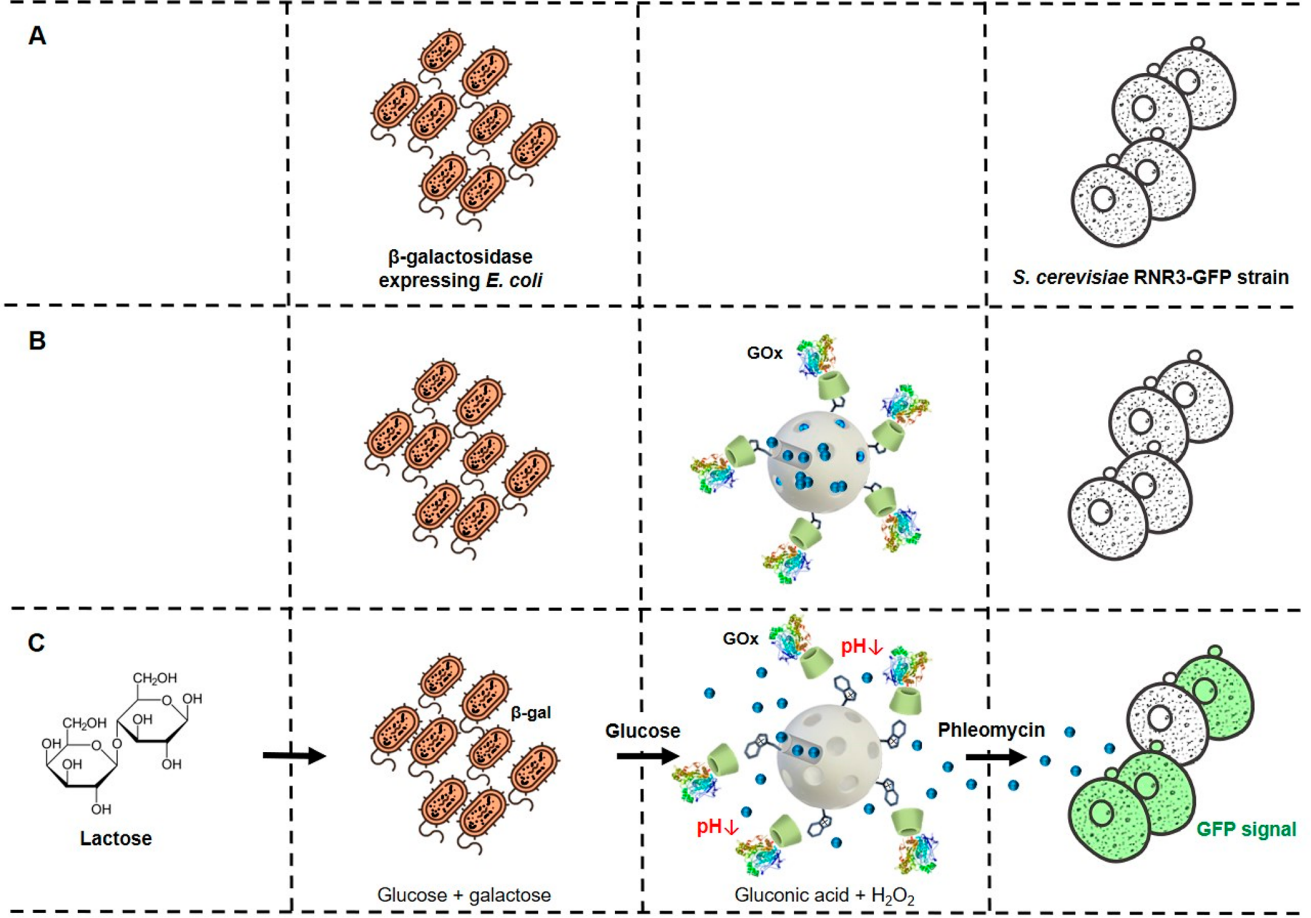
Representation of the Reported Nanoprogrammed Chemical Communication Paradigm between Microorganisms from Different Kingdoms (A) *E. coli* (β-galactosidase-expressing) bacterium cells do not communicate with *S. cerevisiae* yeast cells under normal conditions. (B) Tailor-made mesoporous nanoparticles (loaded with phleomycin and capped with a GOx-based responsive gatekeeper) are added to enable communication. (C) Communication steps: bacterium cells convert lactose into glucose and galactose; glucose (first chemical messenger) is detected by the nanodevice inducing delivery of the entrapped phleomycin (second chemical messenger); finally, the receiver yeast cells sense phleomycin and respond by activating expression of GFP.

Interaction between species in our proposed system is carried out through an aqueous medium by means of chemical communication channels as both microorganisms have cell walls composed of proteins, lipids, and polysaccharides that avoid the internalization of nanoparticles unless specific permeability treatments are applied.^36,37^ The engineered bacteria used in our studies (*E. coli* DH5α) carries a plasmid (pTZ57R) encoding lacZ (β-galactosidase production) and ampicillin resistance. The budding yeast strain employed expresses GFP upon exposure to DNA-damaging agents since its transcription is controlled by the RNR3 promoter.^38^ Accordingly, GFP fluorescence signal is triggered in the presence of a genotoxin such as phleomycin. The “nanotranslator” is based on mesoporous silica nanoparticles due to the advantageous properties they have such as their chemical stability, large loading capacity and the great variety of cargoes which may be entrapped in their pores. Moreover, their surface can be decorated with a wide range of targeting groups, gatekeepers and enzymes showing a stimuli-responsive nature with tailor-made properties for versatile integration in communication scenarios.^39^ In particular, our nanocarrier is based on mesoporous silica nanoparticles functionalized with benzimidazole (Bz) units on the external surface and capped by the formation of an inclusion complex with glucose oxidase-modified β-cyclodextrin (GOx-CD). This pH-sensitive supramolecular gatekeeper disassembles when glucose is present in the surroundings as the enzyme units produce gluconic acid inducing a local drop of pH and causing the protonation of benzimidazole moieties (p*K*_a_ = 5.55);^40^ the disruption of the benzimidazole:β-cyclodextrin complex leads to the uncapping of the pores and the delivery of the entrapped cargo.

To start with, we synthesized and characterized the sensing-actuating nanoparticles (see Supporting Information for details). We first prepared GOx-functionalized nanoparticles loaded with a fluorescent dye ([Ru(bpy)_3_]Cl_2_) as model cargo. Indeed, the resulting nanoparticles had a spherical shape, a size of around 100 nm and a pore network as observed by transmission electron microscopy (Figures 1 and SI-1). In addition, powder X-ray diffraction, N_2_ adsorption–desorption isotherms, dynamic light scattering, elemental analysis, enzymatic assays, and TEM-EDX were used to complete their characterization (Figure SI-2 to SI-6). Then, we tested the ability of the nanodevice to autonomously deliver the entrapped cargo upon exposure to glucose. To do so, we brought dye-loaded GOx-capped nanoparticles (NP_GOx-Dye_) in aqueous solution (1 mg·mL^–1^) at pH 7.5 and monitored cargo delivery in the presence and absence of glucose by measuring the fluorescent signal of the released dye. A clear release was observed in the presence of glucose due to the opening of the GOx-CD-Bz gatekeeper; whereas in contrast, cargo delivery was insignificant in the absence of glucose (Figure SI-7). Moreover, the specificity of the nanodevice was verified by confirming that cargo delivery was not observed in the presence of other saccharides, such as fructose, galactose, lactose, and sucrose (Figure SI-9). After confirming the programmed sensing-actuating behavior, we prepared similar nanoparticles loaded with phleomycin (NP_GOx-Phl_) that would have a receiver–sender role and enable the full communication shown in Scheme 1. We also confirmed that NP_GOx-Phl_ was able to retain phleomycin and deliver it on-command in the presence of glucose (Figure SI-8).

**Figure 1:**
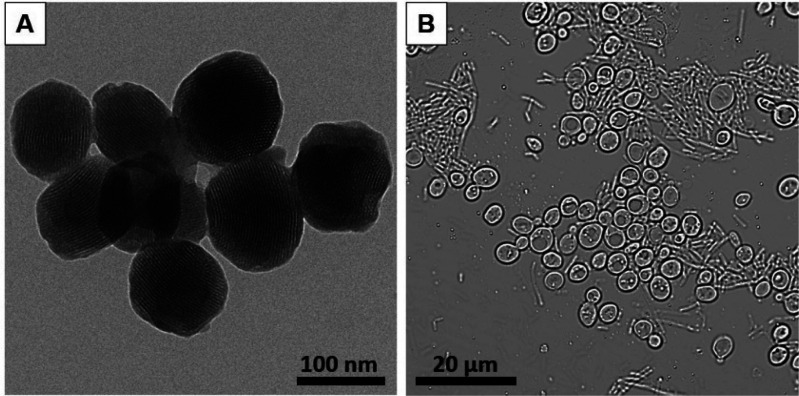
Images of the nanoparticles and microorganisms employed to construct the communication system. (A) Transmission electron microscopy (TEM) image of cargo-loaded GOx-functionalized gated mesoporous silica nanoparticles. (B) Bright-field microscopy image of a coculture of *Escherichia coli* bacterium cells (tubular morphology), and *Saccharomyces cerevisiae* yeast cells (nearly spherical morphology).

As a next step and envisaging the final designed communication system (Scheme 1C), we then checked the response of the selected microorganisms to their corresponding stimulus. First, for assessing the ability of engineered *E. coli* cells to process lactose, β-galactosidase expression was confirmed by qualitative and quantitative enzyme activity assays by means of X-Gal staining and *o*-nitrophenyl-β-d-galactopyranoside hydrolysis in aqueous medium (determined β-galactosidase activity = 8.0 mU·mL^–1^, culture OD = 0.5; see SI Section 13). Moreover, to test the response of yeast cells to phleomycin (chemical message), positive and negative control experiments were carried out by adding or not free phleomycin to yeast culture (at mid log exponential growth phase), that was further incubated for 3 h in the presence of *E. coli*. When coincubated (Figure 1B) and upon visualization by bright-field microscopy (Figure 1B), *S. cerevisiae* yeast cells could be distinguished by their near-spherical shape with a size of around 5 μm, whereas *E. coli* bacterium cells exhibited their characteristic tubular morphology of around 0.5 μm of diameter and 5–10 μm in length. Experiments in the presence of phleomycin (as depicted in Figure SI-12) indeed revealed GFP expression in *S. cerevisiae* yeast cells when coincubated with bacteria for 3 h in fructose-supplemented medium^41^ (as carbon source).

Next, we set out to validate the first linear communication pathway of the network, that is, communication between bacteria (acting as sender) and the nanodevice NP_GOx-Dye_ (acting as receiver). With this aim, we conducted a series of delivery studies in which *E. coli* bacterium cells (4 × 10^9^ cells·mL^–1^) and NP_GOx-Dye_ (1 mg·mL^–1^) were combined in aqueous solution (pH 7.5) in the absence or presence of lactose (2%, as trigger of the communication). As additional control, dye release from NP_GOx-Dye_ in the absence of bacteria and the presence of lactose was also monitored. As plotted in Figure 2, a steady increase in cargo delivery ([Ru(bpy)_3_]Cl_2_) was observed in the complete combination (lactose + bacteria + nanoparticle), whereas no substantial dye release was observed either in the absence of lactose (bacteria + nanoparticle, red curve) or in the presence of lactose and absence of bacteria (lactose + nanoparticle, black curve) (see Table 1). Altogether, this corroborates the establishment of a linear communication model: bacteria are able to hydrolyze lactose (input) and catalyze the formation of glucose, which is sensed by the GOx-capped nanodevice with the subsequent cargo delivery. In the absence of bacteria, the nanodevice is insensitive to lactose as this disaccharide is not recognized by the GOx enzyme.

**Table 1:** Summary of Linear Bacteria–NP_GOx-Dye_ Communication Experiments

condition	input^a^	bacteria^a^	nanodevice^a^	response^a^
a	+	+	+	+
b	–	+	+	–
c	+	–	+	–

a Presence or absence of input (lactose), bacteria and nanodevice is represented by + and – , respectively, whereas response refers to significant (+) or negligible (% <20%) (−) cargo delivery.

**Figure 2:**
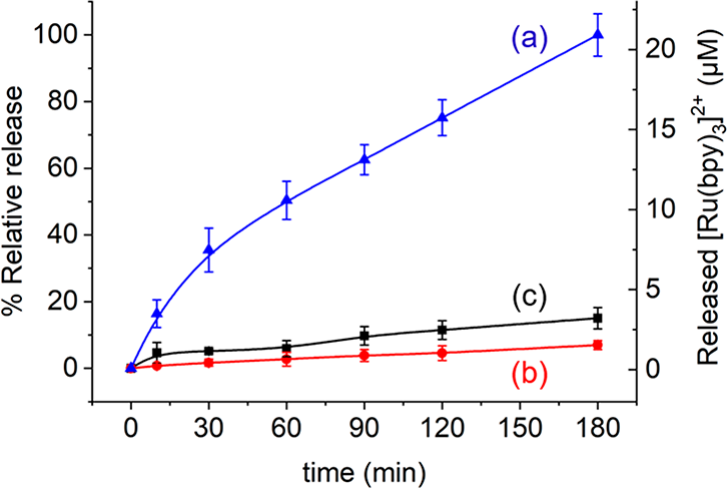
Validation of lactose-responsive linear communication pathway between *E. coli* cells (acting as sender) and the dye-loaded nanodevice NP_GOx-Dye_ (acting as receiver). Kinetics of cargo release ([Ru(bpy)_3_]Cl_2_) in aqueous solution at pH 7.5 containing NP_GOx-Dye_ and bacteria in the absence (b, red curve) and presence (a, blue curve) of lactose (2%). As additional control, release from NP_GOx-Dye_ in the presence of lactose and absence of bacteria was also monitored (c, black curve). Error bars correspond to the s.d. from three independent experiments.

In our subsequent set of experiments, we tested the second linear communication pathway, that is, information transmission from the nanodevice to yeast cells. To do so, yeast cells (1.5 × 10^8^ cells·mL^–1^) were incubated with phleomycin-loaded GOx-capped nanoparticles (NP_GOx-Phl_) in aqueous medium at pH 7.5 containing glucose (2%). As a control, we additionally prepared phleomycin-loaded nanoparticles lacking the GOx enzyme, yet capped with β-cyclodextrin (NP_Phl_), and incubated them with yeast cells under the same conditions. After 3 h of incubation, induction of GFP expression was assessed by confocal fluorescence microscopy. As shown in Figure 3, the micrographs revealed a clearly higher fluorescent signal when yeast cells were incubated with NP_GOx-Phl_ (panel a), as compared to nonfunctional NP_Phl_ (panel b, lacking the enzyme). Quantification of the corresponding images (using ImageJ) revealed an about 5-fold increase in fluorescence upon incubation with functional NP_GOx-Phl_, as compared to control NP_Phl_. In order to address why certain (yet relatively low) GFP emission was observed in the negative control, we performed additional control experiments: (i) with no nanoparticles added but with glucose and (ii) with no nanoparticles but with glucose and the phleomycin equivalent corresponding to the determined background leakage (Figure SI-13). Both of these additional controls showed a low GFP emission similar to the control with nonfunctional NP_Phl_; thus, these experiments suggest that yeast cells exhibit certain background GFP expression under control conditions, yet GFP expression is considerably enhanced upon communication with the functional nanoparticles. Altogether, this confirms the ability of NP_GOx-Phl_ to recognize glucose in the medium and deliver the phleomycin cargo (messenger) that triggers GFP expression in yeast cells. In nanoparticles lacking the GOx enzyme, the communication is disrupted.

**Figure 3:**
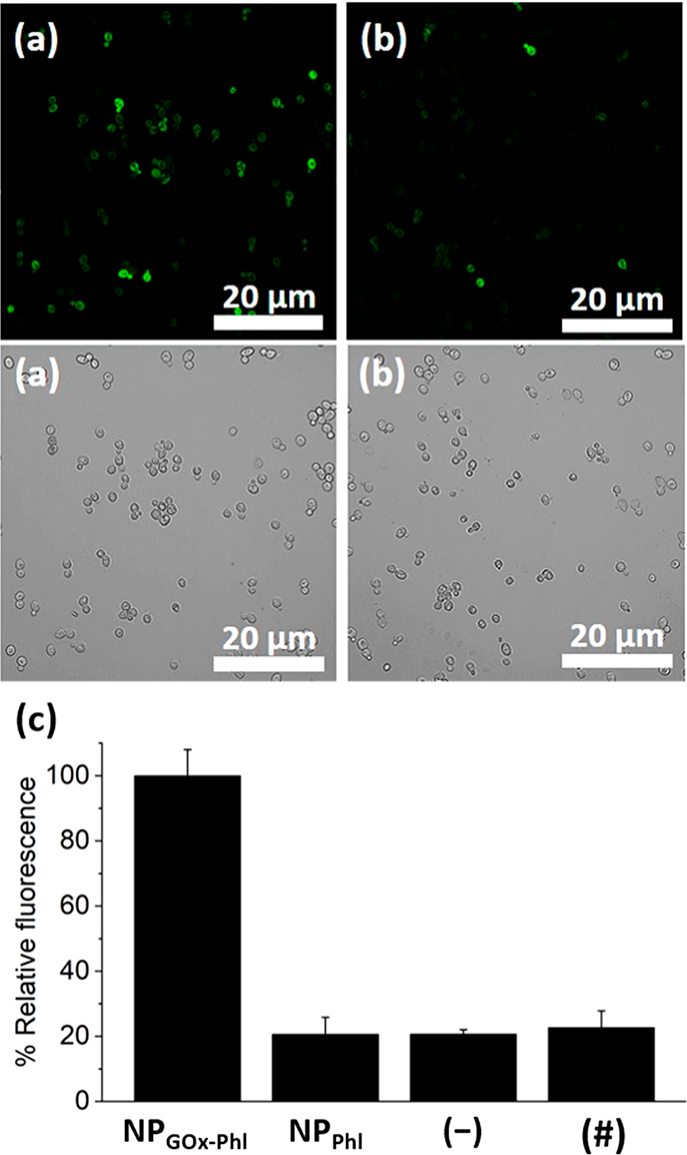
Validation of the glucose-responsive linear communication pathway between the phleomycin-loaded GOx-capped nanodevice NP_GOx-Phl_ (acting as sender) and *S. cerevisiae* yeast cells (acting as receiver). Monitorization of GFP fluorescence in *S. cerevisiae* yeast cells upon incubation with glucose (2%) and (a) phleomycin-loaded GOx-capped nanodevice (NP_GOx-Phl_) or (b) control nanoparticle NP_Phl_ (lacking the GOx enzyme). Top, fluorescence images; bottom, bright-field images. Samples were incubated for 3 h. (c) Normalized quantification of the GFP-associated fluorescence intensity for yeast cells treated with the corresponding nanoparticles or controls. Two percent of glucose was added in all cases. (−) represents control in the absence of nanoparticles and (#) is control in the absence of nanoparticles with the phleomycin equivalent corresponding to the determined background leakage. Data represent mean ± s.e.m. (*n* = 3). Additional images are showed in Figure SI-13.

After validating both linear communication pathways separately, we then constructed the complete nanopro-grammed cross-kingdom communication system. As depicted in Scheme 1, this involves a concatenated flow of information from the bacterium cells to the “nanotranslator” and subsequently to the yeast cells. To setup these experiments, yeast and bacteria were inoculated individually in fresh YPD medium and incubated until reaching mid log exponential phase. Then, both microorganisms were brought together in YPD medium (glucose-free, supplemented with fructose) and mixed with an aqueous solution at pH 7.5 of NP_GOx-Phl_ (50 μg·mL^–1^). Then, 2% of lactose (input of the communication) was added. As control, parallel experiments were carried out with nanoparticles NP_Phl_ (phleomycin-loaded β-cyclodextrin-capped nanoparticles lacking the GOx enzyme). Confocal fluorescence microscope images (Figure 4 and Figure SI-14) showed GFP-associated fluorescence when the “nanotranslator” NP_GOx-Phl_ was present, whereas the fluorescent signal was significantly lower when the uncomplete nanoparticles NP_Phl_ were employed. Quantification of GFP-associated fluorescence intensity from three independent experiments (Figure 4e) revealed more than a 4-fold emission increase in the presence of NP_GOx-Phl_, as compared to the control (i.e., NP_Phl_). As additional control experiments to rule out any potential side interaction, we also prepared unloaded GOx-functionalized nanoparticles (NP_GOx_) and unloaded nanoparticles also lacking GOx (NP_Control_). As expected, significantly lower GFP expression was observed in confocal fluorescence microscopy studies in the same conditions when using NP_GOx_ or NP_Control_, indicating that there is not chemical information flow when the nanoparticles did not contain cargo or/and enzyme. In addition, experiments in which bacteria and yeast cells were incubated in the absence of nanoparticles (see (−) in Figure 4e) showed similar GFP intensity levels as with control nanoparticles, which can be attributed to certain background expression in agreement with previous studies (see Table 2).^38^ Furthermore, we determined the viability of yeast cells after conducting communication experiments based on the quantification of colony formation units (CFUs) after incubation for 24 h; no reduction in cell viability was observed when using nonfunctional NP_Phl_ (lacking the enzyme). In contrast, a remarkable reduction in CFU counts was observed when functional NP_GOx-Phl_ was added, which is ascribed to the genotoxic action of the released phleomycin (Figure SI-16). These experiments demonstrate the hierarchical cross-kingdom communication of bacterium cells with yeasts through the use of an abiotic “nanotranslator” involving the directional exchange of two chemical messengers (glucose and phleomycin). The behavior of this communication network can be expressed in a Boolean logic table of five elements (i.e., the triggering input (lactose), the first microorganism (bacteria), the GOx enzyme on the nanodevice, the phleomycin cargo, and the receiver microorganism (yeast)). Among 32 possible entries (Table SI-4), only the complete system bacteria-NP_GOx-Phl_-yeast leads to effective cross-kingdom communication.

**Table 2:** Summary of Different Experimental Conditions in Communication Studies Involving Bacteria–Nanodevice–Yeast Populations^a^

cond.	bacteria^b^	enzyme^b^	cargo^b^	NP^b^	yeast^b^	output^b^
a	+	–	+	+	+	–
b	+	+	+	+	+	+
c	+	+	–	+	+	–
d	+	–	–	+	+	–
e	+	–	–	–	+	–

a Corresponding with a–d micrographs and quantification in Figure 4.

b Presence or absence of a component is represented by + and–respectively, whereas output refers to significant (+) or negligible (% <25%) (−) GFP signal in receiver yeast cells.

**Figure 4:**
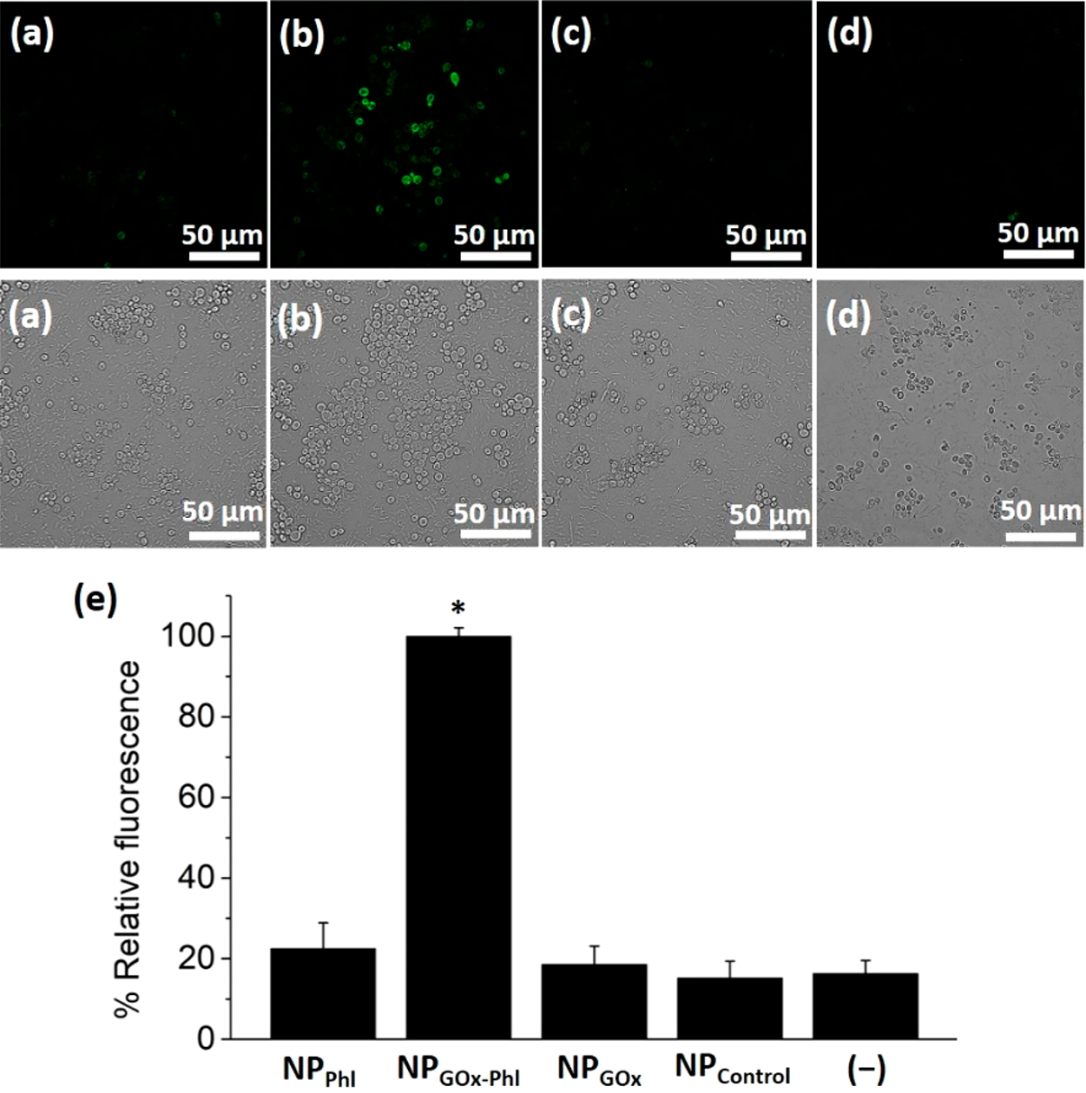
Validation of the nanoprogrammed cross-kingdom cellular communication in mixtures of *E. coli* bacterium cells, nanoparticles, and *S. cerevisiae* yeast cells. Evaluation of fluorescent signal from GFP expression in *S. cerevisiae* yeast cells upon incubation with *E. coli* bacterium cells and nanoparticles under different conditions (summarized in Table 2): (a) with phleomycin-loaded enzyme-lacking nanoparticles (NP_Phl_), (b) with phleomycin-loaded GOx-functionalized “nanotranslator” (NP_GOx-Phl_), (c) with unloaded GOx-functionalized nanoparticles (NP_GOx_), and (d) with unloaded nanoparticles also lacking the GOx enzyme (NP_Control_). Top, fluorescence images; bottom, bright field images. Samples were incubated for 3 h in medium containing 2% lactose (input of the communication). Additional images are provided in the Supporting Information (Figure SI-14 and Figure SI-15). (e) Normalized quantification of the GFP-associated fluorescence intensity for the different experimental conditions. (−) represents control in the absence of nanoparticles (conditions in Table 2). Several fields of view of each condition were analyzed obtaining similar results. Data represent mean ± s.e.m. from thee independent experiments (*p < 0.001).

As an interesting (and so-far underexplored) aspect, spatial information transmission and propagation of sequential actions should be considered when designing chemical communication networks between micro/nanosystems. In an additional set of experiments, we employed microfluidic channels to control the relative spatial location of each communicating entity (bacteria–nanoparticles–yeast). As depicted in Figure 5, the experimental setup consisted of two reservoirs (60 μL) located at opposite ends of a connecting channel (d = 17 mm) that allow the propagation of chemical signals. After filling the channels with YPD medium supplemented with 2% lactose (trigger of the communication), the reservoirs were completed with additional medium and different combinations of communicating entities. In the first condition (a), bacteria and yeast cells were located in opposite reservoirs ([B]—[Y]), as control experiment where information flow would not occur due to the absence of nanoparticles. In the second condition (b), bacteria and nanoparticles were located in the first reservoir and yeast cells in the opposite ([B,N]—[Y]), thus locating the communication action 1 (transmission of glucose from bacteria to nanoparticles) in the first reservoir and the subsequent propagation of the second chemical messenger happening through the channel (transmission of phleomycin from nanoparticles to yeast, communication action 2). In the third condition (c), bacteria were located in the first reservoir, and yeast cells together with nanoparticles in the opposite ([B]—[N,Y]); thus, inducing the transmission of glucose through the channel (from bacteria to nanoparticles, communication action 1) and subsequently, communication step 2 happening in the second reservoir (transmission of phleomycin from nanoparticles to yeast in close proximity). After incubation (15 h), yeast cells were collected and visualized (Figure SI-17). As expected, a relatively low fluorescence was quantified (Figure 5d) for the control experiment ([B]—[Y]). For the second condition ([B,N]—[Y]), the relative fluorescence substantially increased (to ∼70%), which indicated the activation of GFP production due to spatial transmission of information. This represents an about 2.9-fold increase compared to the control, yet this relative increase is smaller than in the bulk experiment (Figure 4e), which indicates slower dynamics that can be ascribed to the propagation of sequential actions when the communicating entities are spatially separated. Interestingly, the third condition ([B]—[N,Y]) showed enhanced activation compared to (b), indicating efficient transmission of information under this spatial arrangement. Together, these experimental observations allow one to point out phleomycin (messenger in action 2) dilution as the main limiting factor in the spatial propagation of information; its dilution through the channel (in the [B,N]—[Y] configuration) results in partially diminished yeast activation, whereas phleomycin release in close proximity to yeast (in the [B]—[N,Y] configuration) results in a more effective activation.

**Figure 5:**
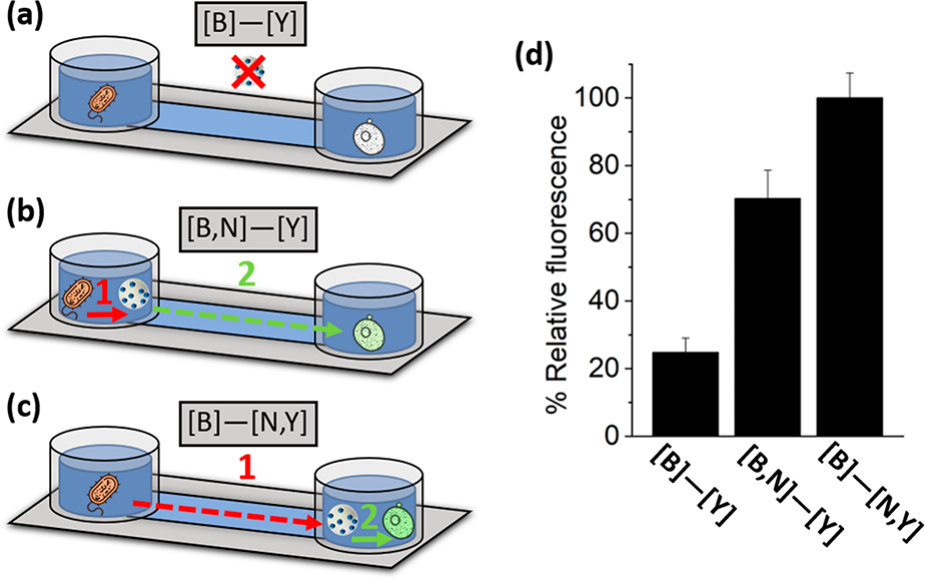
Information transmission under different spatial arrangements. (a–c) Schematics of the experimental setup using microfluidic channels with bacteria and yeast located on opposite reservoirs. Different conditions represent (a) without nanoparticles, (b) nanoparticles in the bacteria’s reservoir, and (c) nanoparticles in the yeast’s reservoir. Arrows represent communication process 1 (transmission of glucose from bacteria to the nanoparticles) and communication process 2 (transmission of phleomycin from the nanoparticles to yeast). (d) Corresponding quantification of the GFP-associated fluorescence intensity for the different experimental conditions. Several fields of view of each condition were analyzed obtaining similar results. Data represent mean ± s.e.m. (*n* = 3).

Overall, the engineered cross-kingdom communication cascade requires the exchange of two chemical messengers and the resulting production of a reporter protein. To better understand the dynamics of our multicomponent system, we decided to compare the relative signals of the two messengers and output signal at the same time scales. Similar communication experiments to as described above in bacteria–nanodevice–yeast mixtures were performed stopping the experiment at different times, that is, at 60, 120, and 180 min (Figure SI-18). As depicted in Figure 6, a relatively low GFP signal was observed after 60 min incubation, which strongly increased at 120 min almost reaching saturation (∼96%). Moreover, no free glucose was detected in the mixture (using a commercial detection kit) at the scheduled times which suggested full consumption of glucose by the nanoparticles. Indeed, spectrophotometric assays (see SI for details) revealed that the rate of glucose production by bacteria (0.0034 μmol min^–1^) is lower than the rate of glucose consumption by the nanoparticles (0.084 μmol min^–1^). This also correlates with the fact that cargo release is slower in the linear lactose-triggered bacteria–nanoparticle communication experiments (Figure 3) as compared to when the nanoparticles are exposed to an equivalent concentration of glucose (Figure SI-8). In the absence of nanoparticles, we determined that the amount of substrate transformed by bacteria follows a linear trend (Figure SI-10), as expected for first-order enzymatic reactions, which can be correlated to the relative signal corresponding to glucose production as showed in Figure 6. For the cargo release, we extracted the relative signal showed in Figure 6 by employing dye-loaded nanoparticles as previously described. Interestingly, at these time points signal 1 (generated glucose) and signal 2 (cargo release) followed a linear relationship (Figure SI-19a), which could be potentially attributed to a coupling between the two signaling processes with glucose generation by bacteria being the limiting step. In contrast, comparison of these data also revealed that signal 3 (GFP intensity) reached saturation faster than signal 2 (cargo release), which indicates effective activation of yeast cells once a certain partial release of cargo (∼75% at 120 min) is reached (Figure SI-19b).

**Figure 6:**
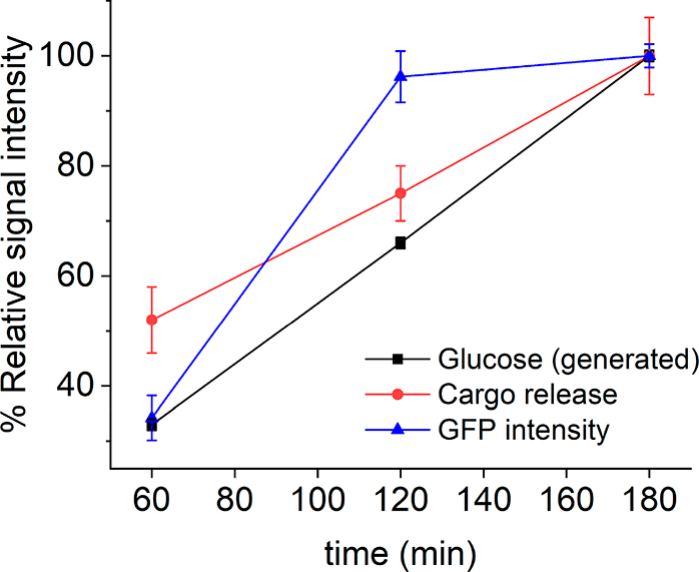
Relative intensity of the different signals involved in the communication process: chemical messengers (glucose and cargo release ([Ru(bpy)_3_]Cl_2_)) and output signal (GFP-associated fluorescence) at different time points.

In summary, we report herein the nanoprogramming of cross-kingdom communication between living microorganisms, which involves two different cells and tailor-made nanoparticles acting as “nanotranslators”. In our proof-of-concept system, molecular information from the environment (lactose) is processed by β-galactosidase-expressing *E. coli* bacteria and transformed into a chemical signal (glucose). Glucose is detected by the nanoparticles; subsequently, the nanoparticles translate the chemical message “glucose” to the chemical messenger “phleomycin” which is understandable for the receiver microorganism (*S. cerevisiae*). In response to phleomycin, *S. cerevisiae* yeast cells activate a genetic cascade that leads to green fluorescent protein expression as the output of the communication. The whole network can be described as two hierarchically concatenated linear communication pathways, that is, bacteria–nanodevice and nanodevice–yeast, which are independently validated. Cross-kingdom communication is demonstrated herein with functional nanoparticles that exhibited a double receiver-sender role, while communication is disrupted when the nanoparticles are incomplete.

This contribution is, as far as we know, the first realization of engineered cross-kingdom cellular communication mediated by nanoparticles and illustrates the potential to design chemical communication pathways at the micro/nanoscale involving several living and abiotic micro/nanosystems. The topic of chemical communication is still in its infancy and proof-of-concept demonstrations are a first necessary step toward the realization of future applications in fields such as biomedicine, microbiology and biotechnology. Whereas we based most of our experiments in standard well-established methods, the development of future applications will require more advanced methodologies to enable monitorization of chemical communication processes in complex settings such as biological tissues.

With development of “nanotranslators” that enable cross-kingdom communication a wide range of applications can be envisioned. For instance, we might communicate messages that instruct cells to halt physiological processes or initiate protective behaviors; designing particles that can enable plants and fungi talk to each other could help us develop new ways to protect plants; while repurposing the finely honed language that some pathogens or cancer cells use to turn off the immune system may be a way to design new treatments for difficult-to-treat diseases. Potentially, nanoparticles could be engineered as “nanokillers” to program the death of certain cells using chemical communication pathways, in fact, we observed the inhibition of yeast proliferation when the communication cascade is established, which as is an interesting area for further research. Ultimately, we envision that the cross-kingdom cellular communication enabled by nanoparticles will provide new therapeutic and diagnostic methods, biotechnological tools, ways to tune cellular behavior, and contribute to further increase our understanding of biological processes.

## Supporting Information Available

Chemicals, general methods, characterization, and additional procedures and figures (PDF)
