# Supplementary material for: Nanoprogrammed Cross-Kingdom Communication Between Living Microorganisms
Source: Nano Lett. 2022 Feb 16;22(5):1836–44. doi: 10.1021/acs.nanolett.1c02435 (PMC9940291; doi:10.1021/acs.nanolett.1c02435)
Supplement: Supplementary file 1 — nl1c02435_si_001.pdf [file nl1c02435_si_001.pdf]

Supporting Information

Nanoprogrammed Cross-Kingdom Communication Between Living Microorganisms

Beatriz de Luis,[†,‡] Ángela Morellá-Aucejo,[†,‡,§] *Antoni Llopis-Lorente,*[†,‡,*] *Javier Martínez-Latorre,*[†,‡] *Félix Sancenón,*[†,‡] *Carmelo López,*[Δ] *José Ramón Murguía,*[†,‡] *and Ramón Martínez-Máñez*[†,‡,¥,§,*]

[†] Instituto Interuniversitario de Investigación de Reconocimiento Molecular y Desarrollo Tecnológico (IDM), Universitat Politècnica de València, Universitat de València, Camino de Vera s/n, 46022 Valencia, Spain.

[‡] CIBER de Bioingeniería, Biomateriales y Nanomedicina (CIBER-BBN), 28029 Madrid, Spain.

[¥] Unidad Mixta UPV-CIPF de Investigación en Mecanismos de Enfermedades y Nanomedicina, Universitat Politècnica de València, Centro de Investigación Príncipe Felipe, 46012 Valencia, Spain.

§ Unidad Mixta de Investigación en Nanomedicina y Sensores, Universitat Politècnica de València, Instituto de Investigación Sanitaria La Fe, 46026 Valencia, Spain.

Δ Instituto Universitario de Conservación y Mejora de la Agrodiversidad Valenciana, Universitat Politècnica de València (COMAV-UPV), 46022 Valencia, Spain.

1. Chemicals

Tetraethyl orthosilicate (TEOS), *n*-cetyltrimethylammonium bromide (CTABr), sodium hydroxide (NaOH), tris(2,2′-bipyridyl)dichlororuthenium(II) hexahydrate $[Ru(bpy)_3]Cl_2 \cdot 6H_2O$, phleomycin from *Streptomyces verticillus*, (3-iodopropyl)trimethoxysilane, benzimidazole, triethylamine, β-cyclodextrin, glucose oxidase from *Aspergillus niger*, D-(+)-glucose, D-(+)-saccharose, D-(+)-galactose, D-(−)-fructose, D-(+)-lactose monohydrate, 2,2′-azino-bis(3-ethylbenzothiazoline-6-sulfonic acid) diammonium salt (ABTS), peroxidase from horseradish (HRP), ampicillin sodium salt, *o*-nitrophenyl-β-D-galactopyranoside, 5-bromo-4-chloro-3-indolyl-β-D-galactopyranoside, glucose assay kit (MAK263), and bovine serum albumin (BSA) were purchased from Sigma-Aldrich. Sodium sulfate anhydrous, sodium dihydrogen phosphate monohydrate, disodium hydrogen phosphate heptahydrate, toluene and acetonitrile were provided by Scharlau. Bacteriological peptone, yeast extract, and Luria-Bertani broth were purchased from Conda Lab. Agar was purchased from Sigma-Aldrich.

2. Microorganisms strains and culture conditions

β-galactosidase-expressing *Escherichia coli* bacteria used in this study were obtained by transforming *E. coli* DH5α with pTZ57R plasmid which encodes for lacZ gene (β-galactosidase production) and ampicillin resistance using standard protocols.[1] Standard methods for bacteria culture and manipulation were used.

RNR3-GFP *Saccharomyces cerevisiae* yeast strain used in this study was purchased from Life Technologies. It was obtained by tagging RNR3 (systematic name YIL066C) open reading frame (ORF) through oligonucleotide-directed homologous recombination.[2] The genotype of the

parent haploid of the *S. cerevisiae* yeast strain used (ATCC 201388) is: MATα his3Δ1 leu2Δ0 met15Δ0 ura3Δ0. Standard methods for yeast culture and manipulation were used.

3. General methods

Powder X-ray diffraction (PXRD), transmission electron microscopy (TEM), N_2 adsorption-desorption isotherms, UV-visible and fluorescence spectrophotometry, dynamic light scattering (DLS) and elemental analysis techniques were employed for materials characterization. PXRD measurements were performed on a Seifert 3000TT diffractometer using CuKα radiation at low angles (1.3 < 2θ < 8.3, with steps of 0.04 degrees and 3 seconds for step). TEM images were acquired using a JEOL TEM-1010 Electron microscope working at 100 kV. Additionally, TEM coupled with energy dispersive X-ray spectroscopy (TEM-EDX) was used for element mapping using a JEOL TEM-2100F microscope. DLS studies were performed using a ZetaSizer Nano ZS (Malvern). N_2 adsorption-desorption isotherms were recorded on a Micromeritics TriStar II Plus automated analyser. Samples were previously degassed at 90 °C in vacuum overnight and measurements were performed at 77 K. UV-visible spectra were recorded with a JASCO V-650 Spectrophotometer. Fluorescence measurements were carried out in a JASCO FP-8500 Spectrophotometer. Elemental analysis was performed using a LECO CHNS-932 Elemental Analyser. Confocal microscopy imaging was performed employing a Leica TCS SPE (Leica Microsystems Heidelberg GmbH) inverted laser scanning confocal microscope using a HC PL APO 40x oil objective.

Confocal and bright field images were employed for determining the relative fluorescence signal corresponding to GFP for each condition (Figure 3, 4 and 5). Images were analysed using *ImageJ*, a well-known software for image quantification. For each micrograph, the number of yeast cells and the corresponding fluorescence for each cell were extracted (using standard *ImageJ* algorithms). Each micrograph fluorescence was determined by counting the number of activated yest cells (mean pixel intensity >40 a.u.) divided by the total number of cells in the image. To normalize the data as expressed in the quantification figures, % Relative fluorescence for each condition was calculated as (mean micrograph fluorescence for the given condition) / (maximum micrograph fluorescence in the corresponding set of experiments) x100.

4. Synthesis of mesoporous silica nanoparticles (MSNPs)

1.00 g (2.74 mmol) of *n*-cetyltrimethylammonium bromide (CTABr) was dissolved in 480 mL of deionized water. Then, the pH was basified by adding 3.5 mL of a 2 mol·L^{-1} NaOH solution and the temperature was increased to 80 °C. Next, tetraethyl orthosilicate (TEOS) (5 mL, 22.4 mmol)

was added dropwise into the solution. Magnetic stirring was kept for 2 hours to give a white suspension. Finally, the solid was isolated by centrifugation, washed several times with water until neutral pH and dried at 70 °C overnight (as-synthesized MSNPs). To obtain the final mesoporous nanoparticles MCM-41 type (**MSNPs**), the as-synthesized solid was calcined at 550 °C in an oxidant atmosphere for 5 hours in order to remove the surfactant.

5. Synthesis of $NP_{GOx\text{-}Dye}$

60 mg of **MSNPs** were suspended in anhydrous acetonitrile (4 mL) and reacted with 60 µL of (3-iodopropyl)trimethoxysilane for 5.5 h. The solid was isolated by centrifugation, washed with acetonitrile and dried at 70 °C overnight. To functionalize the surface with benzimidazole moieties, 0.25 g of benzimidazole and 990 µL of triethylamine were mixed with 20 mL of toluene and heated for 20 min at 80 °C to prepare a saturated solution of benzimidazole. 10 mL of this suspension were added over 60 mg of the previously prepared nanoparticles. The mixture was stirred at 80 °C for three days. Afterward, the benzimidazole-functionalized solid was isolated by centrifugation and washed with toluene and dried at 70 °C overnight yielding **S0** (benzimidazole-functionalized MSNPs). Next, the loading process was carried out by suspending the solid **S0** (25 mg) in 2 mL of a concentrated solution of $[Ru(bpy)_3]Cl_2{\cdot}6H_2O$ in anhydrous acetonitrile ($2 mg{\cdot}mL^{-1}$), and stirred overnight in order to achieve the loading of the pores. Finally, the solid was centrifuged, washed thoroughly with acetonitrile and dried under vacuum. Then, the loaded support (5 mg) was suspended in 2 mL of 50 mM phosphate buffer at pH 7.5 and capped by the addition of 2.5 mg of lyophilized β-cyclodextrin-modified glucose oxidase derivative (GOx-β-CD, synthesized as previously reported)[3] and stirred overnight at 10 °C. Finally, the solid was centrifuged, washed thoroughly with 50 mM phosphate buffer at pH 7.5 and dried under vacuum. This process yielded nanodevice **$NP_{GOx\text{-}Dye}$**, which was kept wet in refrigerator until use.

6. Synthesis of NP_{Dye}

Nanoparticles **NP_{Dye}** were prepared from **S0** following the same procedure described for **$NP_{GOx\text{-}Dye}$** but the capping step was carried out with 2.5 mg of commercial β-cyclodextrin in 2 mL of 50 mM phosphate buffer at pH 7.5. The resulting **NP_{Dye}** was kept wet in refrigerator until use.

7. Synthesis of $NP_{GOx\text{-}Phl}$

Nanodevice **$NP_{GOx\text{-}Phl}$** was prepared following the same procedure described for **$NP_{GOx\text{-}Dye}$** but the cargo loading process was carried out with phleomycin. To do so, 5 mg of **S0** were suspended in 1 mL of a concentrated solution in ultrapure H_2O of the antibiotic phleomycin (10 mg, 6.5 mM)

and stirred overnight at 10 °C. The solid was isolated by centrifugation and washed with cold ultrapure H_2O. The GOx-β-CD capping was conducted following the same procedure described for **$NP_{GOx-Dye}$**. This process finally yielded **$NP_{GOx-PhI}$**, which was kept wet in refrigerator until use.

8. Synthesis of NP_{PhI}

Nanomaterial **NP_{PhI}** was prepared from **S0** following the same procedure described for **$NP_{GOx-PhI}$** but the capping step was carried out with 2.5 mg of commercial β-cyclodextrin in 2 mL of 50 mM phosphate buffer at pH 7.5. The resulting **NP_{PhI}** was kept wet in refrigerator until use.

9. Synthesis of NP_{GOx}

Nanomaterial **NP_{GOx}** was prepared following the same procedure described for **$NP_{GOx-PhI}$** but the mesoporous container was not loaded. The resulting **NP_{GOx}** was kept wet in refrigerator until use.

10. Synthesis of $NP_{Control}$

Nanomaterial **$NP_{Control}$** was prepared following the same procedure described for **NP_{Dye}** but the mesoporous container was not loaded. The resulting **$NP_{Control}$** was kept wet in refrigerator until use.

11. Characterization

Nanomaterials were characterized by standard techniques (see below).

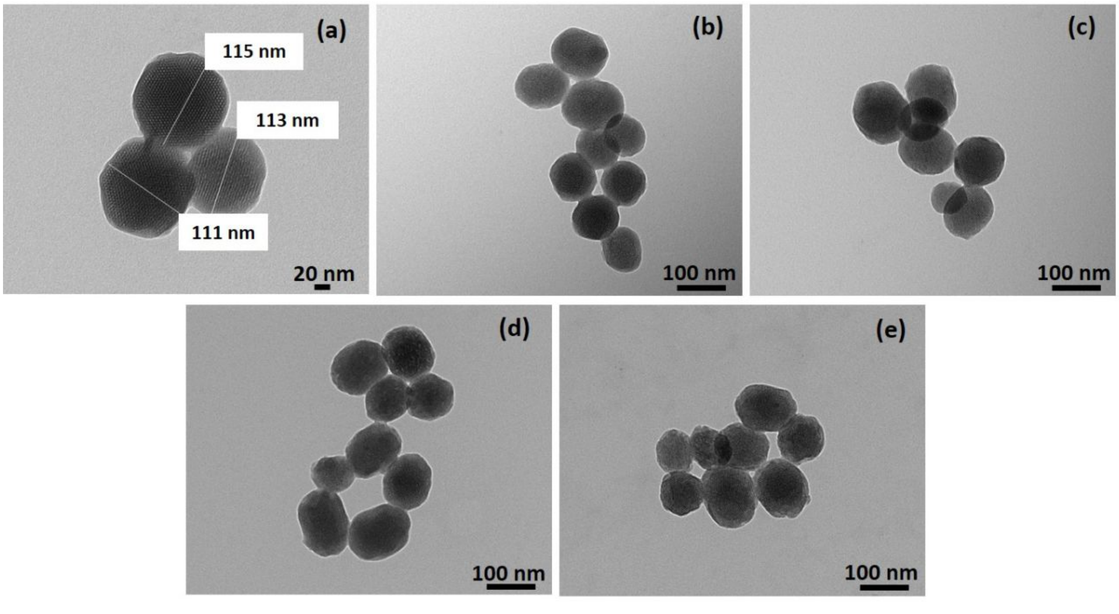

Figure SI-1. TEM images of the calcined mesoporous silica nanoparticles (**MSNPs**) (a-c) and the final nanodevice **$NP_{GOx-Dye}$** (d-e).

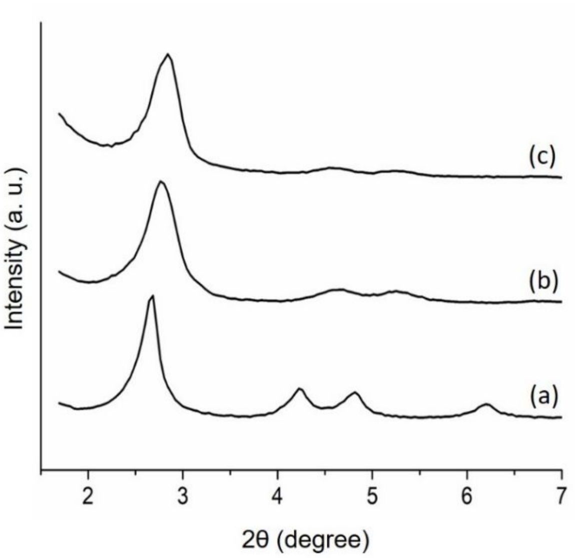

Figure SI-2. Powder X-ray diffraction patterns of the nanomaterials (a) **as-made MSNPs**, (b) **calcined MSNPs** and (c) benzimidazole-functionalized nanoparticles (**S0**).

Figure SI-2 shows powder X-ray diffraction patterns at low angles (1.5 < 2θ < 7). The **as-made MSNPs** (mesoporous silica nanoparticles, a) exhibit characteristic low-angle reflections peaks of MCM-41 type mesoporous scaffolds. For the **calcined MSNPs** (b), we observed a slight displacement of the peaks related to the condensation of silanol groups during the calcination process. These low-angle typical peaks are preserved in the benzimidazole-functionalized nanoparticles (**S0**, c). The presence of the (100) peak in the PXRD patterns in the nanomaterial **S0** indicated that the functionalization had not damaged the mesoporous structure. Powder X-ray diffraction pattern of **$NP_{GOx-Dye}$**, **$NP_{GOx-PhI}$**, **NP_{PhI}**, **NP_{GOx}** and **$NP_{Control}$** were not obtained due to the low quantity collected in the synthesis but neither the enzyme immobilization procedure nor the cargo loading step were expected to affect the mesoporous structure as they were performed under mild conditions.

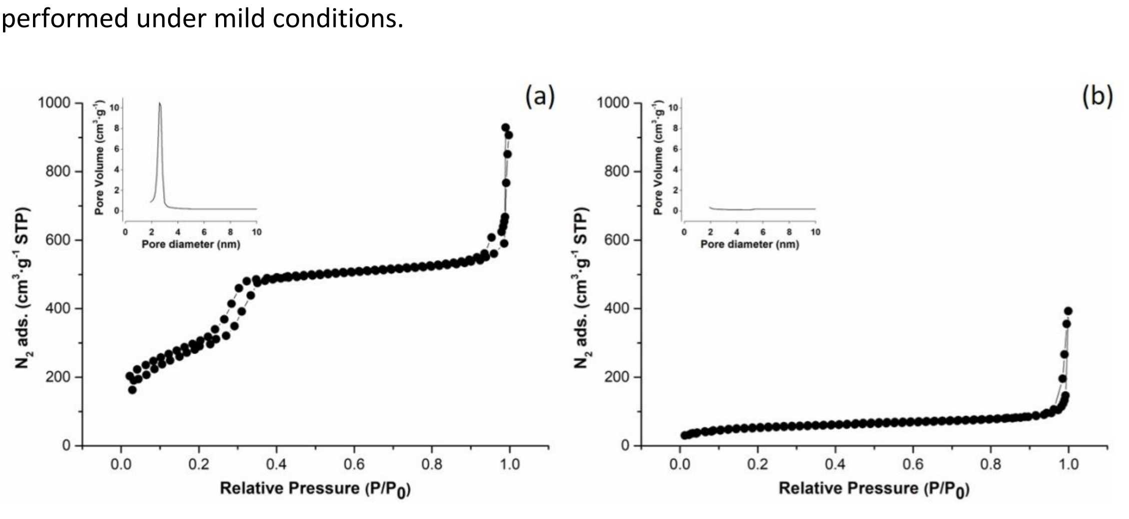

Figure SI-3. The N_2 adsorption-desorption isotherms for (a) the **calcined MSNPs** and the benzimidazole-functionalized nanomaterial (**S0**) loaded with $[Ru(bpy)_3]Cl_2$.

The N_2 adsorption-desorption isotherm of the **calcined MSNPs** shows an adsorption step at intermediate P/P_0 value 0.3, which is characteristic for mesoporous solids with empty pores. This step is related to the nitrogen condensation inside the mesopores by capillarity. The absence of a hysteresis loop in this interval and the narrow BJH pore distribution suggest the existence of uniform cylindrical mesopores. Application of the BET model results in a value for the total specific surface of 1084 $m^2 \cdot g^{-1}$ for **calcined MSNPs**. Total specific surface area for the benzimidazole-functionalized MSNPs (nanomaterial **S0**) loaded with $[Ru(bpy)_3]Cl_2$ significantly decreased to 191 $m^2 \cdot g^{-1}$ due to the cargo loading inside the mesopores. In order to calculate pore size and total pore volume, the BJH model was applied for $P/P_0 < 0.6$ (associated to adsorption inside the pores). N_2 adsorption-desorption isotherm for the functionalized and loaded nanomaterial **S0** shows a significant decrease in N_2 volume adsorbed and is flat when compared (at the same scale) to that from **MSNPs**. This indicates that there is a significant pore blocking accordingly the loading and capping processes. BET specific values, pore volumes and pore sizes calculated from N_2 adsorption-desorption isotherms for both solids are listed in **Table SI-1**.

Table SI-1. BET specific surface values, pore volumes and pore sizes calculated from N_2 adsorption-desorption isotherms for selected nanomaterials.

Solid	S_{BET} [$m^2 \cdot g^{-1}$]	Pore Volume [$cm^3 \cdot g^{-1}$]	Pore size [nm]
**Calcined MSNPs**	1084 ± 1	0.88	2.53
**Loaded S0**	191 ± 3	0.09	-

The hydrodynamic size and zeta potential of different nanoparticles were measured by dynamic light scattering (DLS) studies (**Table SI-2**, **Figure SI-4**). For carrying out the experiments, the corresponding materials were suspended in distilled water at pH 7 at a concentration of 0.01 $mg \cdot mL^{-1}$.

Table SI-2. Zeta potential (mV) and hydrodynamic size (nm) data.

Nanomaterial	Zeta Potential (mV)	Hydrodynamic Size (nm)
**Calcined MSNPs**	-32.5	112
**S0**	-29.8	130
$\mathbf{NP_{Dye}}$	-27.9	158
$\mathbf{NP_{GOx\text{-}Dye}}$	-38.8	284

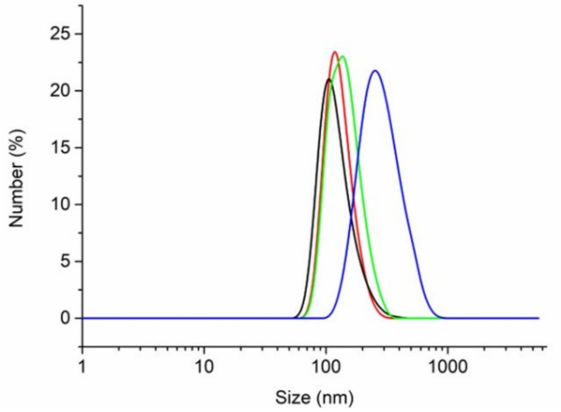

Figure SI-4. Hydrodynamic size (nm) graphic representation of **calcined MSNPs** (black), benzimidazole-functionalized MSNPs (nanomaterial **S0**, red), β-CD-capped and dye-loaded nanoparticles ($\mathbf{NP_{Dye}}$, green) and dye-loaded GOx-β-CD-capped nanodevice $\mathbf{NP_{GOx\text{-}Dye}}$ (blue).

From elemental analysis data (**Table SI-3**), composition of the nanomaterial was estimated. Considering the data obtained from the analysis of the unloaded and uncapped nanoparticles (**S0**, benzimidazole-functionalized MSNPs), the amount of molecular gate $(CH_2)_3$-benzimidazole was determined as 29.0 $mg \cdot g^{-1}$.

Table SI-3. Elemental analysis data.

Nanomaterial	% C	% H	% N
**S0**	4.40	1.34	0.51

The content of phleomycin encapsulated in **$NP_{GOx\text{-}Phl}$** and **NP_{Phl}** was estimated by measuring the absorbance of the phleomycin in solution (λ_{abs} = 300 nm) before and after the capping process as well as all the washing steps until yielding the final nanoparticles. The calibration curve indicated an amount of 231.4 mg of phleomycin per g of **$NP_{GOx\text{-}Phl}$** and 268.1 mg per g of **NP_{Phl}**. The amount of $[Ru(bpy)_3]Cl_2$ loaded in **$NP_{GOx\text{-}Dye}$** was estimated to be 39.8 $mg{\cdot}g^{-1}$ also by calibration. To infer so, the mesoporous scaffold was hydrolysed by incubating the nanoparticles with NaOH 20% at 40 °C for 1 hour and then the supernatant absorbance was measured.

The immobilization of the enzyme was confirmed by running an enzyme activity assay. The method we used in order to test glucose oxidase activity is based on the oxidation of glucose by glucose oxidase which gives gluconic acid and hydrogen peroxide. Then, hydrogen peroxide reacts with ABTS (2,2′-azino-bis(3-ethylbenzothiazoline-6-sulfonic acid) diammonium salt) in the presence of peroxidase (HRP) to form a blue-green product ($ABTS^{+\cdot}$) that can be followed UV-visible spectrophotometry (λ_{abs} = 418 nm).

Reactions for assaying glucose oxidase activity:

$$D\text{-}glucose + O_2 + H_2O \xrightarrow{\text{Glucose oxidase}} D\text{-}gluconic\ acid + H_2O_2$$
$$H_2O_2 + ABTS \xrightarrow{\text{HRP}} ABTS^{\cdot +} + H_2O$$

In a typical experiment, 250 µL of 1 M of glucose (180 $mg{\cdot}mL^{-1}$), 250 µL of ABTS solution (1 $mg{\cdot}mL^{-1}$) and 50 µL of HRP solution (2 $mg{\cdot}mL^{-1}$) were placed in a quartz cuvette. All solutions were prepared in 100 mM sodium phosphate buffer at pH 7.5. Then, 10 µL of either buffer (for blank), commercial enzyme solution in buffer (0.01 $mg{\cdot}mL^{-1}$) or **$NP_{GOx\text{-}Dye}$** (capped with GOx-β-CD and loaded with $[Ru(bpy)_3]Cl_2$), **$NP_{GOx\text{-}Phl}$** (capped with GOx-β-CD and loaded with phleomycin) or **NP_{Phl}** (capped with β-CD and loaded with phleomycin) suspension (5 $mg{\cdot}mL^{-1}$) were added. The mixture was shaken and absorbance at 418 nm was monitored as a function of time. Whereas no change was observed in the absence of nanoparticles or commercial enzyme (blank), a strong blue-green colour appeared in the presence of those. The increase in absorbance ($ABTS^{+\cdot}$ formation) as a function of time in the presence of **$NP_{GOx\text{-}Dye}$**, **$NP_{GOx\text{-}Phl}$** or **NP_{Phl}** and the commercial enzyme solution is depicted in **Figure SI-5**.

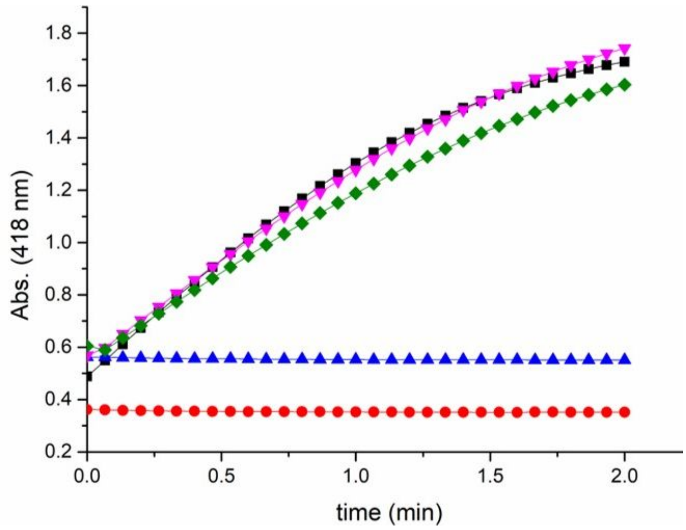

Figure SI-5. Absorbance at 418 nm (ABTS$^{+\cdot}$ formation) due to glucose oxidase activity on **NP$_{GOx\text{-}Dye}$** (pink), **NP$_{GOx\text{-}PhI}$** (green), **NP$_{PhI}$** (blue), commercial enzyme solution (black) and blank (red).

Glucose oxidase activity on the nanoparticles was calculated by applying the following formula:

$$\frac{Enzyme\ Units}{g} = \frac{(\Delta - \Delta_{blank}) \cdot V_T \cdot F_D}{\mathcal{E}_{ABTS} \cdot 1 \cdot V_S \cdot C_S}$$

Where, Δ is the slope of the graph (min^{-1}), Δ_{blank} is the slope of the graph for the blank (min^{-1}), V_T is the total volume in the cuvette, F_D is the dilution factor, $\mathcal{E}_{ABTS}$ is the molar extinction of ABTS at 418 nm (36000 M^{-1} · cm^{-1}), L is the optical path in the cuvette (1 cm), V_S is the volume of the sample added (mL) and C_S is the concentration of sample added (g · mL^{-1}).

The activity of glucose oxidase on **NP$_{GOx\text{-}Dye}$** and **NP$_{GOx\text{-}PhI}$** was determined to be 1.90 U and 1.67 U per mg of solid, respectively, whereas the activity of commercial glucose oxidase was determined to be 125.06 U per mg of commercial enzyme. From this data, the corresponding amount of glucose oxidase on **NP$_{GOx\text{-}Dye}$** and **NP$_{GOx\text{-}PhI}$** was estimated to be 15.16 and 13.34 mg of enzyme per g of solid, respectively.

TEM-EDX mapping of nanodevice **NP$_{GOx\text{-}Dye}$** shows the presence of nitrogen atoms in the whole scaffold. This is attributed to the immobilized enzyme (glucose oxidase) as well as the benzimidazole moiety of the molecular gate (see **Figure SI-6**).

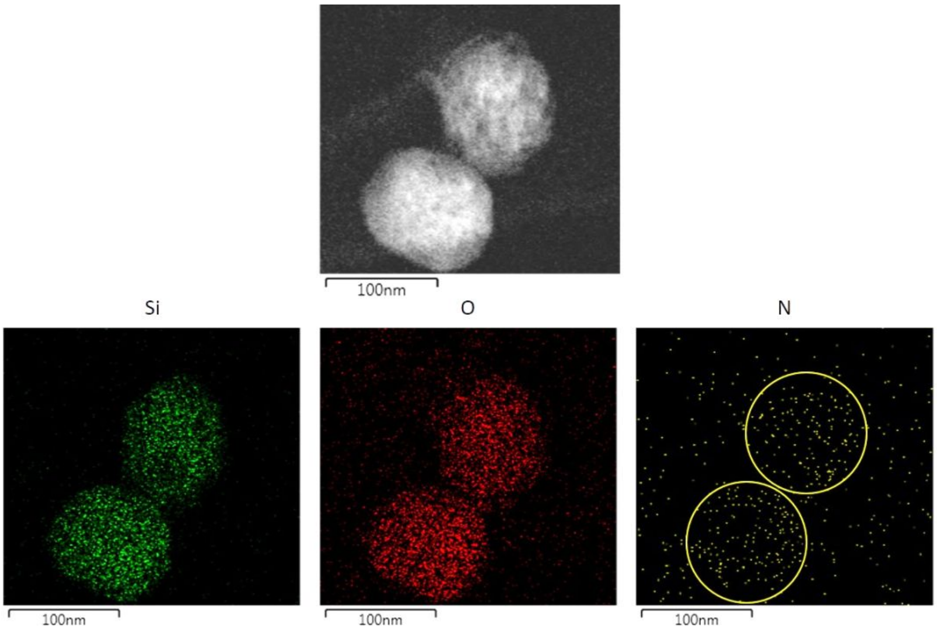

Figure SI-6. TEM-EDX element mapping of nanodevice **NP**$_{\mathbf{GOx\text{-}Dye}}$.

12. Nanodevice sensing-actuating behaviour

Responsive delivery studies

For studying the glucose-responsive release behaviour of **NP**$_{\mathbf{GOx\text{-}Dye}}$, the corresponding refrigerated solution of nanoparticles was washed with an aqueous solution (pH 7.5, 20 mM Na_2SO_4), divided into two fractions and brought to a final concentration of 1 mg·mL^{-1}. Both fractions were incubated for 1 hour. Then, either an aqueous aliquot (for blank) or glucose (1 mM, as input) were added (time = 0 minutes) and samples were shaken over time. Aliquots were taken at scheduled times, centrifuged (2 minutes, 12000 rpm) to remove the nanoparticles and the fluorescence of the solution at 595 nm was measured (λ_{exc} = 453 nm) to determine the relative cargo release.

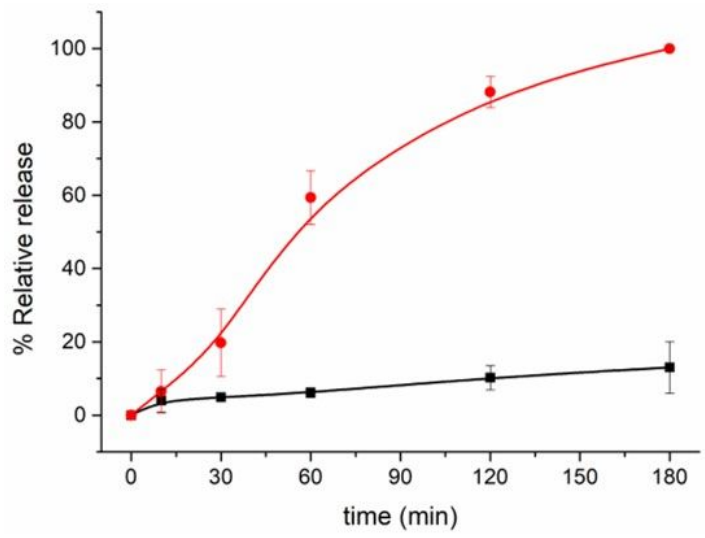

Figure SI-7. Relative cargo release from $\mathbf{NP_{GOx\text{-}Dye}}$ in the absence of input (black curve) and in the presence of glucose (red curve). Substrate added at 1 mM final concentration. Error bars correspond to the s. d. from three independent experiments.

For release experiments with $\mathbf{NP_{GOx\text{-}Phl}}$, the corresponding refrigerated solution of nanoparticles was washed with ultrapure water (pH 7.5), divided into two fractions and brought to a final concentration of 2.5 mg·mL^{-1}. Both fractions were incubated for 1 hour. Then, either ultrapure water (for blank) or glucose (as input) were added (time = 0 minutes) and samples were shaken over time. Aliquots were taken at scheduled times, centrifuged to remove the nanoparticles (2 minutes, 12000 rpm) and the absorbance at 300 nm of the solution measured (phleomycin λ_{abs} = 300 nm).

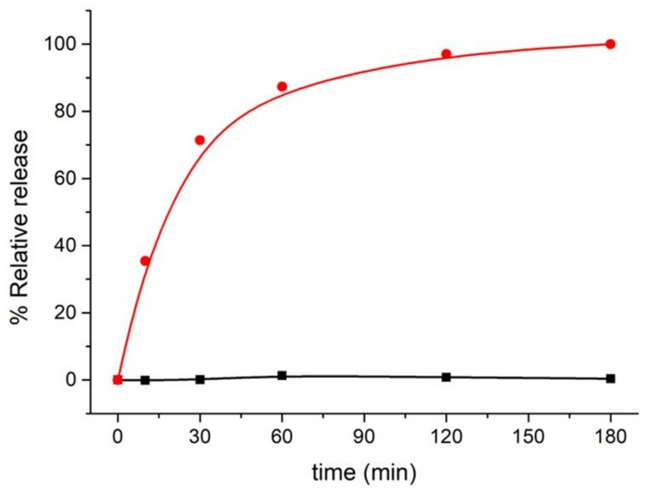

Figure SI-8. Relative cargo release from $\mathbf{NP_{GOx\text{-}Phl}}$ in the absence of input (black curve) and in the presence of glucose (red curve). Substrate added at 1 % final concentration (55.5 mM).

Selectivity in cargo delivery

A refrigerated solution of **NP$_{GOx\text{-}Dye}$** was washed with an aqueous solution (pH 7.5, 20 mM Na_2SO_4), divided into six fractions and brought to a final concentration of 1 mg·mL^{-1}. All fractions were incubated for 1 hour. Then, different monosaccharides (fructose, galactose, glucose) and disaccharides (lactose, sucrose) were added (time = 0 minutes) and samples were shaken over time. Aliquots were taken after 3 hours, centrifuged to remove the nanoparticles (2 minutes, 12000 rpm) and the fluorescence of the solution at 595 nm measured (λ_{exc} = 453 nm) to determine the relative cargo release. A selective response to glucose was observed.

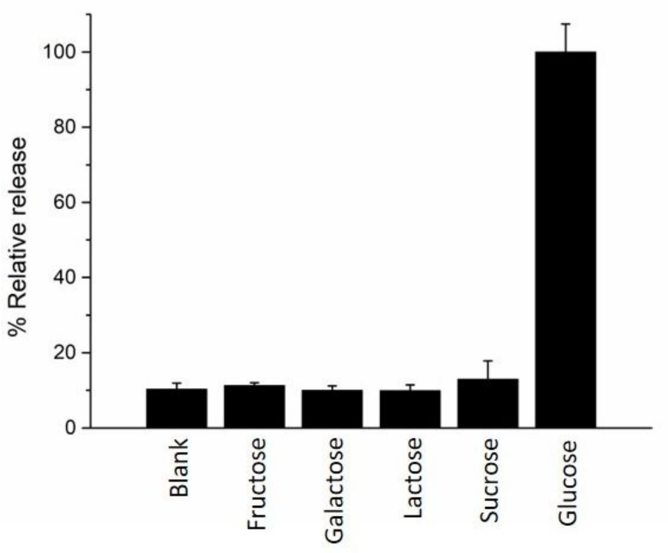

Figure SI-9. Relative cargo release from **NP$_{GOx\text{-}Dye}$** in the presence of different monosaccharides and disaccharides at 1 mM concentration. Error bars correspond to the s. d. from three independent experiments.

13. Cellular experiments

Bacterial β-galactosidase activity assay

The β-galactosidase activity of the engineered *E. coli* cells was confirmed by running a specific enzyme activity assay. The method employed is based on the hydrolysis of *o*-nitrophenyl-β-D-galactopyranoside by β-galactosidase which produces a yellow product (*o*-nitrophenol) that can be followed UV-visible spectrophotometry (λ_{abs} = 420 nm).

Reaction for assaying β-galactosidase activity:

$$o\text{–}nitrophenyl\text{–}\beta\text{–}D\text{–}galactopyranoside \xrightarrow{\text{β-Galactosidase}} o\text{–}nitrophenol + \beta\text{–}D\text{–}galactose$$

To run the assay, mid-exponential *E. coli* transformed with pTZ57R plasmid were grown in LBA (Luria-Bertani medium containing 50 µg·mL^{-1} of ampicillin) shaking at 37 °C until reaching an optical density of 0.5 at 600 nm.

600 µL of *o*-nitrophenyl-β-D-galactopyranoside solution (1.1 mg·mL^{-1}) in ultrapure water at pH 7.5 were placed in a quartz cuvette. Then, 400 µL of either water (for blank) or bacterial culture (previously washed two times with ultrapure water) were added. The mixture was shaken and absorbance at 420 nm was monitored as a function of time. Whereas no change was observed in the absence of bacteria (blank), a yellow colour appeared in the presence of those. The increase in absorbance (*o*-nitrophenol formation) as a function of time in the presence of β-galactosidase-expressing *E. coli* is depicted in **Figure SI-10**.

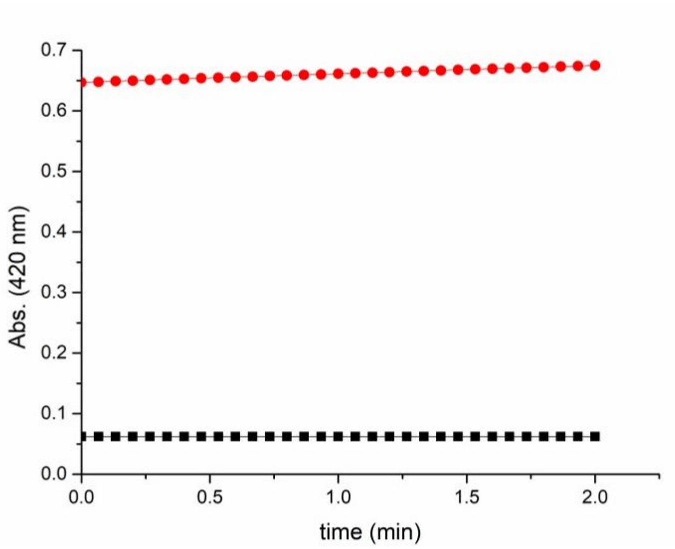

Figure SI-10. Absorbance at 420 nm (*o*-nitrophenol formation) due to β-galactosidase activity on *E. coli* cells (red) and blank (black).

Bacterial β-galactosidase activity was calculated by applying the previously explained formula (page S10) considering that the molar extinction of *o*-nitrophenol ($\mathcal{E}_{ONP}$) is 4500 M^{-1}· cm^{-1} at 420 nm. The activity of β-galactosidase was determined to be 0.0077 U per mL of bacterial culture.

In addition, β-galactosidase activity was also confirmed by X-Gal staining. To so do, 40 µl of 5-bromo-4-chloro-3-indolyl-β-D-galactopyranoside (X-Gal, 20 mg·ml^{-1}) in dimethylformamide was spread on LBA agar plates and let to dry. In parallel, *E. coli* cells were grown in LBA medium to an optical density of 0.5 at 600 nm, spread on the previously prepared X-Gal LBA agar plates and incubated at 37 °C for 30 hours.

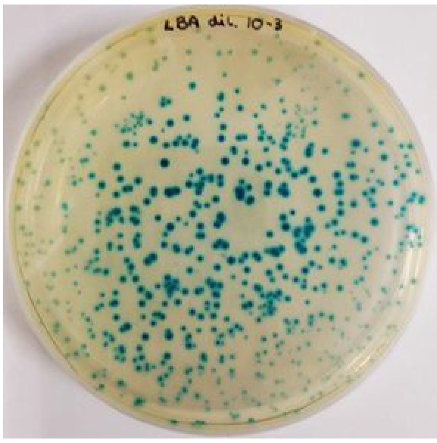

Figure SI-11. Observation of blue *E. coli* colonies after X-gal staining. The insoluble blue product formed after the dimerization of the hydrolysis products of bromo-4-chloro-3-indolyl-β-D-galactopyranoside (5-bromo-4-chloro-3-hydroxy-1H-indole) confirmed β-galactosidase activity in *E. coli.*

Studies of GFP-expression in yeasts-bacteria co-cultures

Intracellular overexpression of green fluorescent protein (GFP) in yeasts was monitored by confocal fluorescence microscopy. In particular, the images were acquired employing a Leica TCS SPE inverted laser scanning confocal microscope using a HC PL APO 40x oil objective with an excitation wavelength of 488 nm. Two-dimensional pseudo-colour images (255 colour levels) were gathered with a size of 1024x1024 pixels and Airy 1 pinhole diameter.

Experiments with free phleomycin (positive and negative GFP controls)

S. cerevisiae budding yeasts RNR3-GFP were inoculated into fresh YPD (yeast extract peptone dextrose) medium and cultured for 3.5 hours at 28 °C. β-galactosidase-expressing *E. coli* bacteria were inoculated into fresh LBA (Luria-Bertani medium containing 50 $\mu g \cdot mL^{-1}$ of ampicillin) and incubated for 2.5 hours at 37 °C. Both were shaken until reaching the log phase with an optical density (600 nm) of approximately 0.5, respectively.

For yeast control experiment, an aliquot of the freshly inoculated yeast suspension was washed three times with glucose-free YPD and resuspended in diluted glucose-free YPD (10 %, pH 7.5). For yeast-bacterium co-culture control experiments, aliquots of 2 mL of the freshly inoculated yeast suspension and aliquots of 2 mL of the freshly inoculated bacteria suspension were mixed, concentrated, washed three times with glucose-free YPD and resuspended in diluted YPD (10 %, pH 7.5). Medium dilution was done with ultrapure water (pH 7.5) and lactose (2 %) was also added. In total, four different conditions were studied: (a) yeast culture, (b) yeast-bacterium co-culture, (c) yeast-bacterium co-culture in the presence of phleomycin (2.6 µM, 4 $\mu g \cdot mL^{-1}$), and

(d) yeast-bacterium co-culture in the presence of phleomycin (2.6 μM, 4 μg·mL^{-1}) supplemented with fructose (1 %). Samples were incubated in continuous shaking for 3 hours at 28 °C. Finally, the samples were centrifuged (5 minutes, 6000 rpm), washed two times with ultrapure water (pH 7.5) and the pellet was resuspended in 40 μL of ultrapure water. Then, fluorescent GFP-associated signal was evaluated by confocal fluorescence microscopy (Figure SI-12). The registered fluorescent signals revealed the need for supplementing the aqueous media with an extra carbon source (fructose) to trigger effective GFP expression in yeasts (as they do not process lactose and glucose production by bacteria is relatively slow). As reported, transcription-translation processes require sufficient energy supply for activation. As expected, a clear increase in GFP-associated yeast fluorescence was observed upon the addition of phleomycin to yeast-bacterium co-culture in fructose-supplemented medium (Figure 12-d).

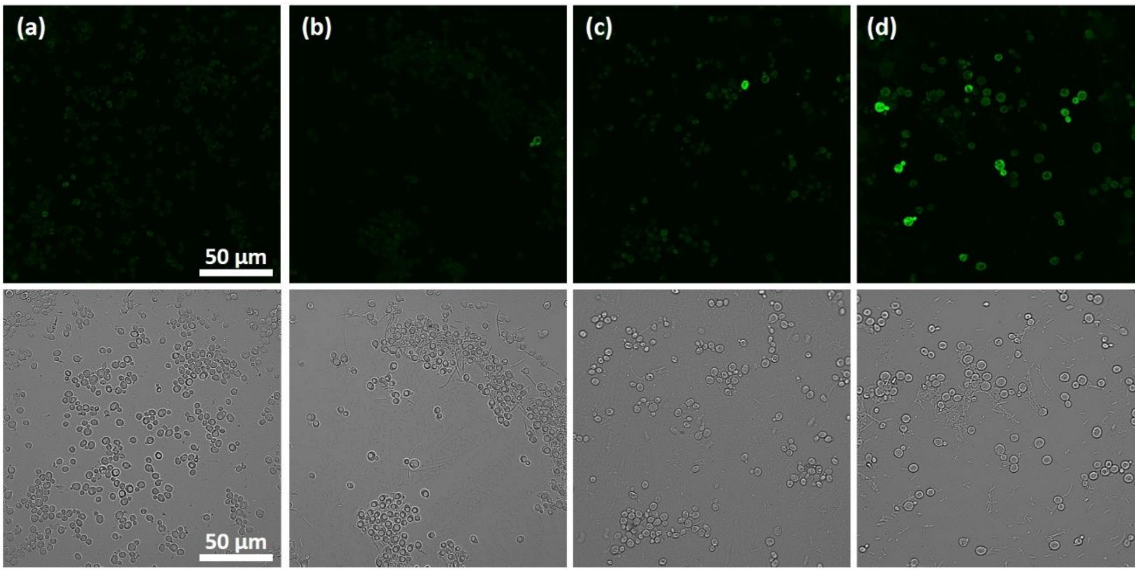

Figure SI-12. Evaluation of GFP signal induction in *S. cerevisiae* cells examined by confocal fluorescence microscopy after 3 hours in aqueous medium (glucose-free 10 % YPD with 2% lactose) under different conditions. (a) Yeast culture (negative control experiment). (b) Yeast-bacterium co-culture (negative control experiment). (c) Yeast-bacterium co-culture in the presence of phleomycin (2.6 μM). (d) Yeast-bacterium co-culture in the presence of phleomycin (2.6 μM) supplemented with fructose (1 %). Top: fluorescence images, bottom: bright field images.

Validation of lactose-responsive linear communication pathway between *E. coli* cells and $\mathbf{NP_{GOx\text{-}Dye}}$

A refrigerated solution of $\mathbf{NP_{GOx\text{-}Dye}}$ was washed with ultrapure water (pH 7.5), divided into different fractions and brought to a final concentration of 1 mg·mL^{-1}. In parallel, 2 mL of a mid-

exponential β-galactosidase-expressing *E. coli* culture (optical density 0.5 at 600 nm) grown in LBA at 37 °C were washed with ultrapure water (pH 7.5) and concentrated to $4{\cdot}10^9$ cells·mL^{-1} in each of the corresponding fractions. **$NP_{GOx\text{-}Dye}$** and bacteria fractions were combined and incubated for 1 hour and then, either ultrapure water (for blank) or lactose (2 %, as input) were added (time = 0 minutes). In parallel, release from **$NP_{GOx\text{-}Dye}$** in the presence of lactose and absence of bacteria was also monitored. Samples were shaken at 28 °C and aliquots were taken at scheduled times, centrifuged (5 minutes, 6000 rpm) and then the fluorescence of the solution at 595 nm was measured (λ_{exc} = 453 nm) to determine the relative cargo release. The obtained kinetics profile is plotted in Figure 2 of the main text.

Validation of the glucose-responsive linear communication pathway between the $NP_{GOx\text{-}Phl}$ and *S. cerevisiae* yeast cells

The refrigerated solutions of **$NP_{GOx\text{-}Phl}$** and **NP_{Phl}** were washed with ultrapure water (pH 7.5), divided into two fractions and brought to a final concentration of 10 µg·mL^{-1}. In parallel, 2 mL of a mid-exponential RNR-GFP *S. cerevisiae* culture (optical density 0.5 at 600 nm) grown in YPD at 28 °C were washed with ultrapure water (pH 7.5), concentrated to $1.5{\cdot}10^8$ cells·mL^{-1} and added to **$NP_{GOx\text{-}Phl}$** and **NP_{Phl}** suspensions. Then, 2 % glucose was added to the mixtures and shaken at 28 °C for 3 hours. Finally, samples were centrifuged (5 minutes, 6000 rpm), washed two times with ultrapure water (pH 7.5) and the pellets were resuspended in 35 µL of ultrapure water. Finally, fluorescent GFP-associated signal was evaluated by confocal fluorescence microscopy (Figure 3, main text). Additionally, following the same procedure, we also performed additional controls with (a) no nanoparticles added but with glucose and (b) no nanoparticles but with glucose and the phleomycin equivalent corresponding to the determined background leakage from **$NP_{GOx\text{-}Phl}$**.

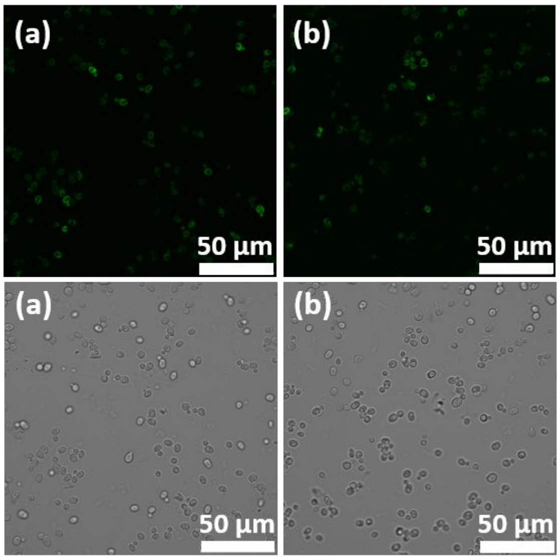

Figure SI-13. Control experiments. Representative confocal microscopy images of GFP signal in *S. cerevisiae* cells after 3 hours for (a) no nanoparticles added but with glucose and (b) no nanoparticles but with glucose and the phleomycin equivalent corresponding to the determined background leakage. Corresponding comparative quantification is showed in Figure 3 of the manuscript.

Validation of the nano-programmed cross-kingdom communication between living microorganisms

S. cerevisiae budding yeasts RNR3-GFP were inoculated into fresh YPD (yeast extract peptone dextrose) and cultured for 3.5 hours at 28 °C. β-galactosidase-expressing *E. coli* bacteria were inoculated into fresh LBA and incubated for 2.5 hours at 37 °C. Both were kept in continuous shaking until reaching the log phase with an optical density (600 nm) of approximately 0.5, respectively.

Aliquots of 2 mL of the freshly inoculated yeast suspension and aliquots of 2 mL of the freshly inoculated bacteria suspension were mixed, concentrated, washed three times with glucose-free YPD and resuspended in glucose-free YPD at pH 7.5. In parallel, the refrigerated solutions of nanoparticles $\mathbf{NP_{GOx\text{-}Phl}}$ and $\mathbf{NP_{Phl}}$ were washed three times with ultrapure water (pH 7.5), divided into the two fractions where yeasts and bacteria have been already added and brought to a final concentration of 50 µg·mL^{-1}. Media were supplemented with 1 % of fructose (final concentration). Then, an input of lactose (2 % final concentration, 55.5 mM) was added to each of the samples followed by incubation under continuous shaking for 3 hours at 28 °C. Finally,

samples were centrifuged (5 minutes, 6000 rpm), washed two times with ultrapure water (pH 7.5) and the pellet was resuspended in 40 µL of ultrapure water. Intracellular overexpression of green fluorescent protein (GFP) was measured and quantified. Several fields of view for each condition in three independent experiments were registered obtaining similar results. Quantification of the fluorescence intensity was determined using ImageJ software.

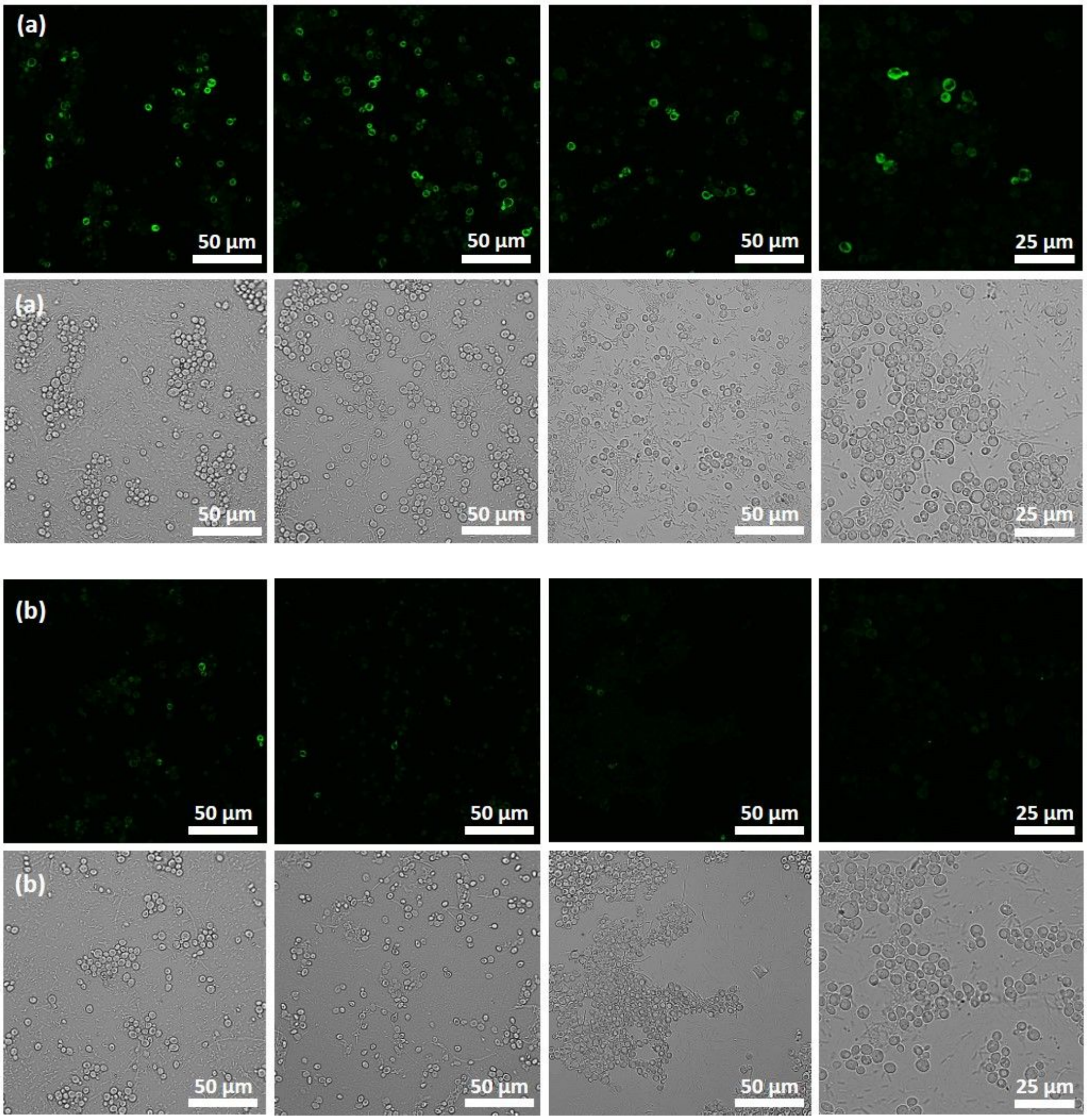

Figure SI-14. Validation of the nano-programmed cross-kingdom communication in mixtures of *E. coli* bacterium cells, nanoparticles and *S. cerevisae* cells. Representative confocal microscopy images of GFP signal induction in *S. cerevisiae* cells in bacteria-yeasts co-cultures after 3 hours for different conditions. *S. cerevisiae* yeast cells were incubated with (a) *E. coli* bacterium cells and phleomycin-loaded GOx-capped nanotranslator (**$NP_{GOx\text{-}Phl}$**); and (b) *E. coli* bacterium cells and control nanoparticles (**NP_{Phl}**, lacking GOx enzyme). Top: fluorescence images, and bottom: bright field images. Samples were incubated for 3 hours in medium containing 2 % lactose (input of the communication).

Additional control experiments with incomplete nanoparticles

The same procedure followed as described in the previous sections was carried out but using non-loaded GOx-functionalized nanoparticles (**NP$_{Gox}$**), and non-loaded GOx-lacking nanoparticles (**NP$_{Control}$**).

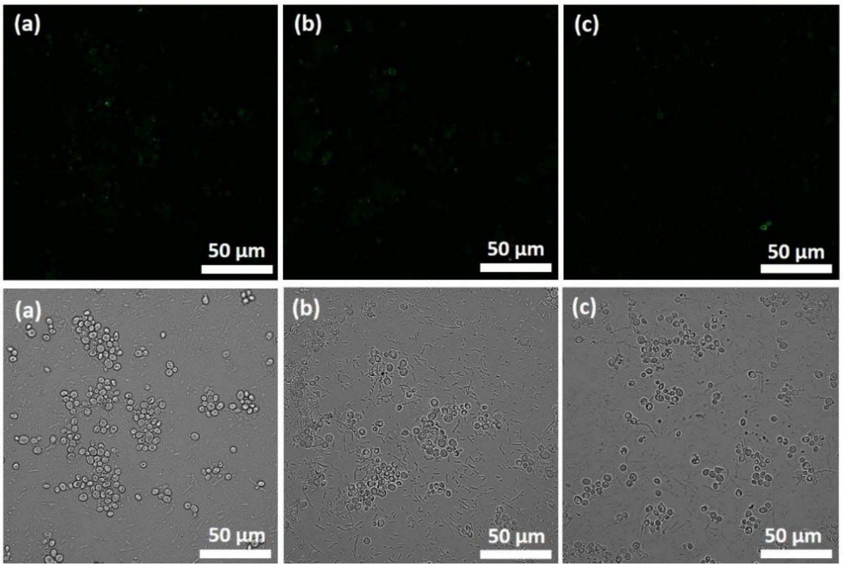

Figure SI-15. Additional control experiments. Representative confocal microscopy images of *S. cerevisiae* cells in bacteria-yeasts co-cultures after 3 hours for (a) incubation without nanoparticles, (b) incubation with unloaded GOx-functionalized nanoparticles (**NP$_{Gox}$**), and (c) incubation with unloaded GOx-lacking nanoparticles (**NP$_{Control}$**). Lactose 2 % was added in each mixture. Top: fluorescence images, and bottom: bright field images.

Yeast cell viability assays

Yeast, bacteria and nanoparticles (**NP$_{GOx-Phl}$**, **NP$_{Phl}$** or without nanoparticles) were incubated for three hours in the presence of lactose (2 %) as described above in cross-kingdom communication experiments. Afterward, samples were centrifuged (5 minutes, 6000 rpm) and washed two times with ultrapure water (pH 7.5). Samples were seeded in YPD agar plates. After incubation for 24 h at 28 °C, colony formation units were quantified by microscopic examination.

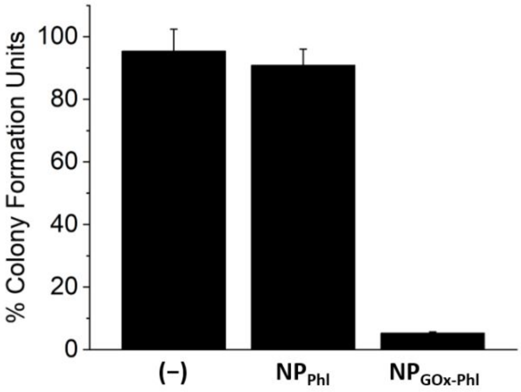

Figure SI-16. Yeast cell viability assays based on the quantification of colony formation units in

cross-kingdom communication experiments under different conditions. Samples were incubated without nanoparticles (-), with **NP_{Phl}** (without enzyme), and with functional **$NP_{GOx\text{-}Phl}$** at a concentration of 50 µg·mL^{-1} in the presence of lactose 2 %. Error bars correspond to the s.e.m. from three independent experiments.

Table SI-4. Summary of the behaviour of the communication system in Boolean logic terms. Presence of a component is represented as 1 and absence as 0. In the presence of the input (1, lactose), the output signal (1) is only produced when all components are present. The output signal is considered 0 when the normalized quantification of the GFP-associated fluorescence intensity is less than 25 % of that found for the complete systems, that is, Input 1, Bacteria 1, Enzyme 1, Cargo 1 and Yeasts 1.

Entry	Input	Bacteria	Enzyme	Cargo	Yeasts	Output
1	0	0	0	0	0	0
2	0	0	0	0	1	0
3	0	0	0	1	0	0
4	0	0	0	1	1	0
5	0	0	1	0	0	0
6	0	0	1	0	1	0
7	0	0	1	1	0	0
8	0	0	1	1	1	0
9	0	1	0	0	0	0
10	0	1	0	0	1	0
11	0	1	0	1	0	0
12	0	1	0	1	1	0
13	0	1	1	0	0	0
14	0	1	1	0	1	0
15	0	1	1	1	0	0
16	0	1	1	1	1	0
17	1	0	0	0	0	0
18	1	0	0	0	1	0
19	1	0	0	1	0	0
20	1	0	0	1	1	0
21	1	0	1	0	0	0
22	1	0	1	0	1	0
23	1	0	1	1	0	0
24	1	0	1	1	1	0
25	1	1	0	0	0	0
26	1	1	0	0	1	0
27	1	1	0	1	0	0
28	1	1	0	1	1	0
29	1	1	1	0	0	0
30	1	1	1	0	1	0
31	1	1	1	1	0	0
32	1	1	1	1	1	1

Spatial transmission of information in a microfluidic device

For studying spatial transmission of information, we employed a 6-channel µ-slide from Ibidi (VI 0.5 glass bottom), as represented in Figure 5 in the manuscript. These experiments were performed under sterile conditions. Channels were loaded with 1 mg·mL^{-1} BSA solution in Milli-Q and incubated (>15 min) to passivate the surfaces, followed by washing with Milli-Q. Then, the device was filled with glucose-free YPD medium with 2 % of lactose (also supplemented with 1 % fructose). Yeast cells, bacteria and nanoparticles were treated as described in previous

communication experiments and then 20 µL of the each solution (or 20 µL of aqueous solution for blanks) were added on the corresponding chamber, while keeping the slide flat on horizontal position. The microchannels were incubated for 15 hours in a humid atmosphere (in a preti dish with a tissue wetted with Mili-Q at the bottom, to prevent water evaporation from the chambers, as described by Qian et al.[4]). After 15 hours, yeast cells were taken from their chamber, centrifuged (5 minutes, 6000 rpm), washed two times with ultrapure water (pH 7.5) and the pellet was resuspended in 40 µL of ultrapure water. Then, GFP-associated fluorescence was quantified as previously described.

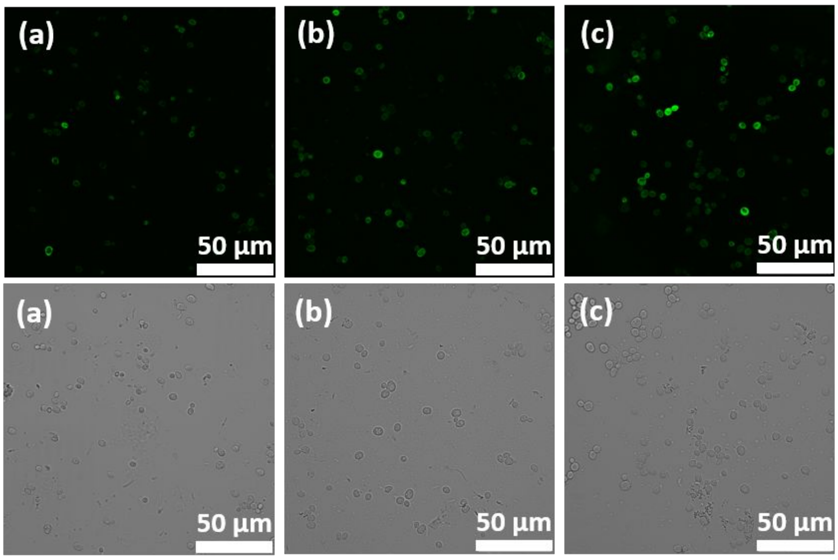

Figure SI-17. Information transmission under different spatial arrangements. Representative confocal microscopy images of *S. cerevisiae* cells after incubation in microfluidic channel experiments, corresponding to Figure 5 in the main manuscript. Different conditions represent incubation (a) without nanoparticles, (b) nanoparticles in the bacteria's reservoir, and nanoparticles in the yeast's reservoir. Top: fluorescence images, and bottom: bright field images.

Nano-programmed cross-kingdom communication between living microorganisms at different time points

The same procedure followed as described in the previous sections was carried out. After shaking the bacteria-yeast-nanoparticle mixtures (in the presence of lactose 2 %) for 60, 120 and 180 min; the samples were centrifuged, washed and visualized under the confocal microscope.

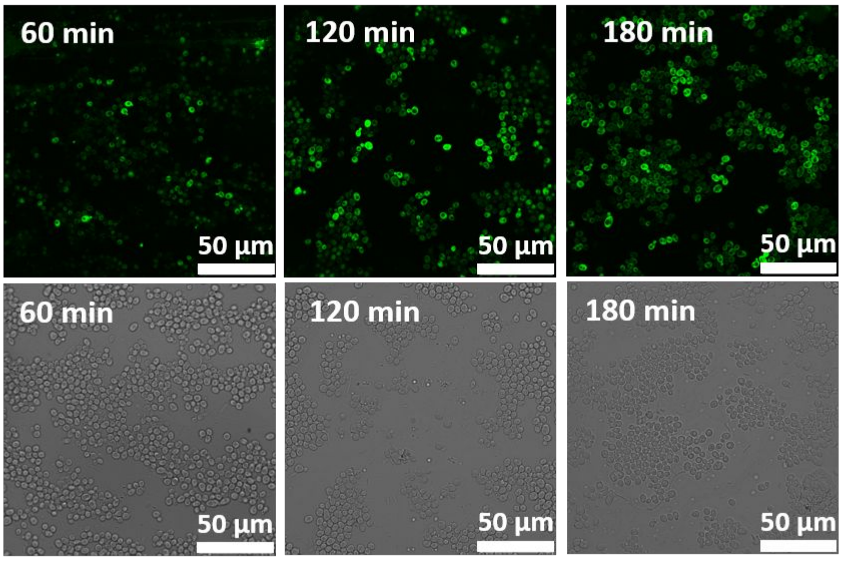

Figure SI-18. Nano-programmed cross-kingdom communication at different time points. Representative confocal microscopy images of *S. cerevisiae* cells in bacteria-yeasts co-cultures with phleomycin-loaded GOx-capped nanotranslator (**NP**$_{\mathbf{GOx\text{-}Phl}}$); after 60, 120 and 180 min. Top: fluorescence images, and bottom: bright field images.

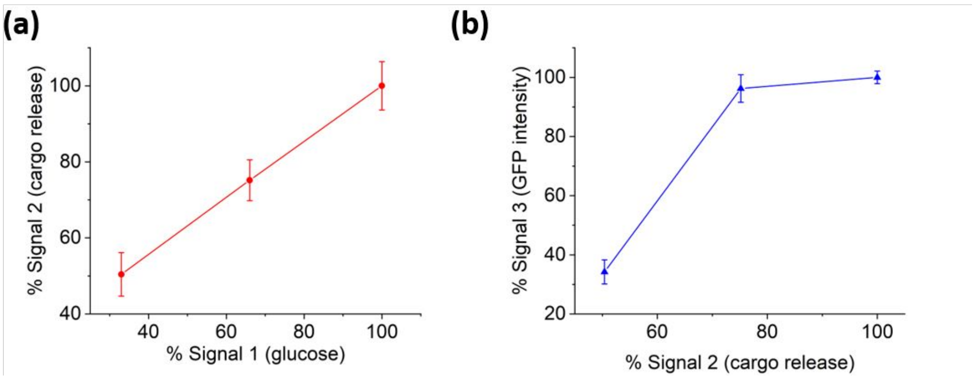

Figure SI-19. Comparative representation of the different signals involved in the overall communication process: (a) signal 2 (cargo release) *vs.* signal 1 (glucose), and (b) signal 3 (GFP intensity) *vs.* to signal 2. Data corresponds to the relative values determined at 60, 120 and 180 min.

16. Supplementary References

[1] J. Sambrook, E. R. Fritsch and T. Maniatis in *Molecular Cloning: A Laboratory Manual,* 2nd ed., Cold Spring Harbor Laboratory Press, NY, USA, **1989**.

[2] W-K. Huh, J.V. Falvo, L. C. Gerke, A. S. Carroll, R. W. Howson, J-S. Weissman and E. K. O'Shea, *Nature* **2003**, *425*, 686-691.

[3] a) E. Aznar, R. Villalonga, C. Giménez, F. Sancenón, M. D. Marcos, R. Martínez-Máñez, P. Díez, J. M. Pingarrón and P. Amorós, *Chem. Commun.* **2013**, *49*, 6391-6393; b) M. Holzinger, L.

Bouffier, R. Villalonga and S. Cosnier, *Biosens. Bioelectron.* **2009**, *24*, 1128-1134; c) K. Hamasaki, H. Ikeda, A. Nakamura, A. Ueno, F. Toda, I. Suzuki and T. Osa, *J. Am. Chem. Soc.* **1993**, *115*, 5035-5040.

[4] X. Qian, I. N. Westensee, C. C. Fernandes, and B. Stadler, *Angew. Int. Ed.* **2021**, 34, 18704-18711.
